# Association between Common Variation in 120 Candidate Genes and Breast Cancer Risk

**DOI:** 10.1371/journal.pgen.0030042

**Published:** 2007-03-16

**Authors:** Paul D. P Pharoah, Jonathan Tyrer, Alison M Dunning, Douglas F Easton, Bruce A. J Ponder, SEARCH Investigators

**Affiliations:** 1 Department of Oncology, University of Cambridge, Cambridge, United Kingdom; 2 Strangeways Research Laboratory, Department of Public Health and Primary Care, University of Cambridge, Cambridge, United Kingdom; University of Michigan, United States of America

## Abstract

Association studies in candidate genes have been widely used to search for common low penetrance susceptibility alleles, but few definite associations have been established. We have conducted association studies in breast cancer using an empirical single nucleotide polymorphism (SNP) tagging approach to capture common genetic variation in genes that are candidates for breast cancer based on their known function. We genotyped 710 SNPs in 120 candidate genes in up to 4,400 breast cancer cases and 4,400 controls using a staged design. Correction for population stratification was done using the genomic control method, on the basis of data from 280 genomic control SNPs. Evidence for association with each SNP was assessed using a Cochran–Armitage trend test (*p*-trend) and a two-degrees of freedom χ^2^ test for heterogeneity (*p*-het). The most significant single SNP (*p*-trend = 8 × 10^−5^) was not significant at a nominal 5% level after adjusting for population stratification and multiple testing. To evaluate the overall evidence for an excess of positive associations over the proportion expected by chance, we applied two global tests: the admixture maximum likelihood (AML) test and the rank truncated product (RTP) test corrected for population stratification. The admixture maximum likelihood experiment-wise test for association was significant for both the heterogeneity test (*p* = 0.0031) and the trend test (*p* = 0.017), but no association was observed using the rank truncated product method for either the heterogeneity test or the trend test (*p* = 0.12 and *p* = 0.24, respectively). Genes in the cell-cycle control pathway and genes involved in steroid hormone metabolism and signalling were the main contributors to the association. These results suggest that a proportion of SNPs in these candidate genes are associated with breast cancer risk, but that the effects of individual SNPs is likely to be small. Large sample sizes from multicentre collaboration will be needed to identify associated SNPs with certainty.

## Author Summary

The polygenic model of cancer susceptibility suggests that multiple alleles contribute to the excess familial risk of most common cancers. Candidate gene association studies have been a commonly used approach in the search for such alleles. We have investigated over 700 common variants in genes that are candidates for breast cancer susceptibility in a large case-control study of breast cancer, but no single variant was identified at an appropriate level of statistical significance. The purpose of this study was to consider these data as a whole, using a novel method, the admixture maximum likelihood test, to test the hypothesis that a proportion (unknown) of the variants we investigated are associated with breast cancer. After adjusting for population substructure, we found evidence for association that was robust to all but the most extreme assumptions about the degree of population stratification. Genes in the cell-cycle control and steroid hormone metabolism and signalling pathways were the main contributors. These results suggest that a proportion of single nucleotide polymorphisms (SNPs) in these candidate genes are associated with breast cancer risk, but that the effects of individual SNPs are likely to be small. Large sample sizes from multicentre collaboration will be needed to identify associated SNPs with certainty.

## Introduction

Breast cancer tends to cluster in families, with disease being approximately 2-fold more common in first-degree relatives of cases [1]. The higher rate of most cancers in the monozygotic twins of cases than in dizygotic twins or siblings suggests that most of the familial clustering is the result of inherited genetic variation rather than lifestyle or environmental factors [2]. Some of this clustering occurs as part of specific familial breast cancer syndromes where disease results from single alleles conferring a high risk. However, such alleles are rare in the population, and highly penetrant variants of *BRCA1* and *BRCA2* account for less than 20% of the genetic risk of breast cancer with other rarer high penetrance genes such as *TP53, ATM,* and *PTEN* counting for less than 5% [3]. Despite extensive efforts, linkage studies have failed to map further more *BRCA*-like highly penetrant cancer susceptibility genes [4]. Together with data on patterns of familial occurrence of cancer that exclude cases because of known high-risk genes, this argues strongly that most genetic susceptibility results from the combined effects of many genetic variants, each of which have a modest effect individually [5]. Family-based linkage studies have been the foundation for the many successes in mapping of genes associated with Mendelian disorders, but they lack power to detect alleles conferring moderate risks that are likely to be the norm in complex disease. The main alternative to linkage studies for disease gene mapping is the association study, in which the frequency of a genetic variant in diseased individuals (cases) and individuals without the disease (controls) are compared [6,7]. Association studies for disease genes are generally based on the “common variant: common disease” hypothesis [8]. Allelic association is present when the distribution of genotypes differs in cases and controls. Such an association provides evidence that the locus under study, or a neighbouring locus, is related to disease susceptibility.

Considerable research effort has been put into the search for low to moderate penetrance breast cancer susceptibility alleles over the past ten years. Most early association studies were based on testing candidate functional polymorphisms in candidate genes, but recently more emphasis has been placed on an empirical approach in which a minimal set of “tagging” single nucleotide polymorphisms (SNPs) that efficiently captures all the common genetic variation in a gene is assayed [9]. In addition, high throughput genotyping technologies have made it possible to assess multiple candidate genes. The analysis of such studies inevitably involves a large number of statistical tests, and there has been much debate about how to analyse the totality of such data, and, in particular, how (or indeed whether) to carry out a correction for multiple hypothesis testing. Most approaches to this problem have considered this as a hypothesis-testing problem, in which the aim is to control the overall “experiment-wise” type I error. Thus, the null hypothesis is that there is no association between the disease and any SNPs in the set, and the aim is to test whether this global null hypothesis of no association can be rejected. A variety of methods has been proposed to test the global null hypothesis [10–18]. Recently we developed a novel method, the admixture maximum likelihood (AML) test, which estimates both the proportion of associated SNPs and their typical effect size [19]. We compared the power of the AML method with several previously proposed approaches by simulation and found that the maximum likelihood approach performed similarly to or better than all other tests across a wide range of scenarios for the alternative hypothesis. The rank truncated product (RTP) method also had good power, though somewhat inferior to the maximum likelihood approach in most cases. A simple Bonferroni correction performed best only when the number of associated SNPs was small.

We have been carrying out association studies in breast cancer for over a decade, and over the past five years most of our work has focussed on a comprehensive tagging approach in candidate genes using a two-stage study design. We now have data from up to 4,400 cases and 4,400 controls on 710 common variants in 117 candidate genes. Here we evaluate the evidence for associations between this set of SNPs and breast cancer using the AML and RTP methods

## Results

**Figure 1:**
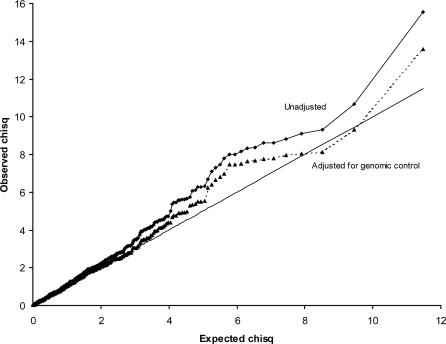
Q–Q Plot for Association of 710 SNPs in Candidate Genes with Breast Cancer Based on First Stage Data

Data were available for 710 SNPs in 120 genes. Genotype frequencies for cases and controls are shown in Table S3. Based on the trend test for association, 53 SNPs (7.5%) were significant at the 5% level, 17 (2.4%) at the 1% level, and one (0.15%) at the 0.1% level. Only one SNP, in the estrogen receptor α gene (trend test χ^2^ = 15.6, *p* = 8 × 10^−5^) reaches the *p* < 0.0001 level, which has been suggested an appropriate threshold for candidate gene studies [9]. However, it failed to reach this threshold after adjusting for population stratification by genomic control (*p*-trend adjusted = 0.00023). Nor was it significant at the 5% level after adjustment for multiple testing using a permutation test that allows for correlation between SNPs tested (the equivalent of a Bonferroni correction for independent hypotheses). Figure 1 shows the Q–Q plots for the univariate trend test using only the first case-control set. The Q–Q plot based on the test statistics adjusted for genomic control follows the line of equivalence for the first 600 SNPs and then starts to deviate as would be expected if a modest proportion of SNPs were associated with disease.

The AML experiment-wise test for association was significant for both the heterogeneity test (*p* = 0.0031) and the trend test (*p* = 0.017), but no association was observed using the RTP method for either the heterogeneity test or the trend test (*p* = 0.12 and *p* = 0.24; respectively). Table 1 shows the results of the AML experiment-wise tests for the complete set of SNPs and for sets of SNPs categorised according to gene functional group. The test for overall association was significant for SNPs in the cell-cycle control genes (*p*-heterogeneity = 0.019, *p*-trend = 0.035), steroid hormone signalling and metabolism genes (*p*-heterogeneity = 0.010, *p*-trend = 0.0080), and the heterogeneous set of genes categorised as “other” (*p*-heterogeneity = 0.0068, *p*-trend = 0.12).

**Table 1:**
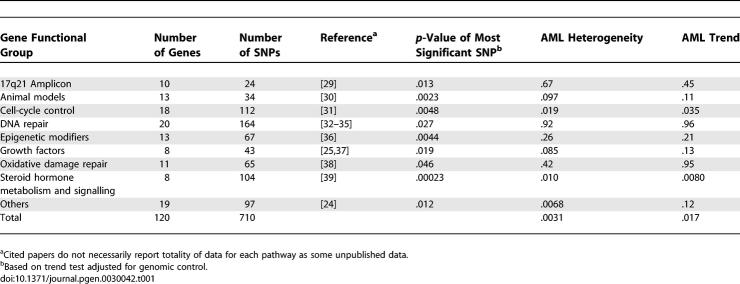
Results of the Experiment-Wise Tests for 710 Candidate Gene SNPs by Gene Functional Group

We reanalysed the data after excluding the most significant SNP (*ESR1* rs3020314) and two SNPs (*CASP8* rs1045485 and *TGFB1* rs1982073), which have been confirmed as being associated with breast cancer in pooled data from up to 20 studies in the Breast Cancer Association Consortium [20]. After excluding these SNPs the test for both heterogeneity and trend remained significant (*p* = 0.0046 and *p* = 0.031, respectively). We also reanalysed the data after removing all 80 tSNPs in these genes. Only the heterogeneity test remained significant (*p* = 0.015, *p*-trend = 0.12). The test for the steroid hormone metabolism pathway remained significant after removing the most significant single SNP (*p*-heterogeneity = 0.018, *p*-trend = 0.012) and all SNPs in *ESR1* (*p*-heterogeneity = 0.10, *p*-trend = 0.044). The significance of the tests for association of “other” genes became borderline after removing the most single significant SNP in *CASP8* (*p*-heterogeneity = 0.0065, *p*-trend = 0.13) and after removing all three SNPs in *CASP8* (*p*-heterogeneity = 0.0058, *p*-trend = 0.12).

Data from the genomic control SNPs indicate some evidence of inflation of the test statistics. Given that λ, the measure of bias due to population stratification, is estimated with error, we repeated the AML tests assuming more extreme levels of bias. We used the estimate of the variance (σ^2^) and the inflation parameter (λ) to carry out sensitivity analyses with the inflation parameter set at λ + σ and λ + 2σ. The test for heterogeneity remained significant for an inflation factors of λ + σ (*p* = 0.012), but not for the most extreme estimate of the bias (inflation parameter = λ + 2σ, *p* = 0.060). The AML trend test was not significant under either scenario (*p* = 0.070 and *p* = 0.20, respectively). Other methods to deal with population stratification have been suggested, each of which has some advantages and disadvantages. The method of structured association uses the genomic control SNPs to assign individuals to specific subpopulations, which can then be used in stratified analyses. This method works well for a small number of discrete of subpopulations but less well for more complex population structures [21]. Gorroochurn and colleagues suggested that the χ^2^_test_ statistics come from a noncentral χ^2^ distribution, and that the noncentrality parameter can be estimated directly using the genomic control SNPs [22]. We adapted this method and estimated the noncentrality parameter (η) by maximum likelihood. The AML tests for association obtained using this method were similar to those used in the genomic control method (data not shown).

It is possible that our results are biased because of systematic differences in genotype frequencies between the incident and prevalent cases (survival bias). We therefore carried out a case only analysis to compare genotype frequencies in incident and prevalent cases. There was no evidence of association using the AML test (*p* = 0.36).

## Discussion

In the past five years we have evaluated over 700 common genetic variants in 120 genes that are candidates for breast cancer susceptibility in a case-control study of over 4,400 cases and 4,400 controls. Based on univariate analyses no definite susceptibility alleles have emerged from this research effort. Only one SNP significant at *p* < 0.0001 was identified in this dataset, and this became less significant after adjusting for population stratification. This association was not significant at a nominal *p* < 0.05 after adjusting for multiple testing. However, it is not clear that this is an appropriate adjustment in such studies, because the result is then highly dependent on the number of SNPs that happen to have been typed at a given time.

The lack of evidence of association in these SNPs may indicate that these genes we have studied do not harbour any susceptibility variants. An alternative explanation is that one or more of the SNPs is associated with disease, but we do not have sufficient statistical power to detect these with appropriate highly stringent levels of statistical significance. For individual variants, the statistical power of the study depends on the at-risk allele frequency, the risks conferred, and the genetic model. For example, assuming that the causative SNP is tagged with *r* ^2^ = 0.8, a type I error rate of 10^−5^, and genotyping success rate of 0.95, the staged study has 67% power to detect a dominant allele with a minor allele frequency (MAF) of 0.05 with an odds ratio of 1.5 or 69% power to detect a dominant allele with MAF of 0.25 with an odds ratio of 1.3. Power to detect recessive alleles is less −39% for an allele with MAF of 0.25 and an odds ratio of 1.5 and 46% for an allele with MAF 0.5 and an odds ratio of 1.3.

We have recently shown that methods that take into account the totality of the data have greater power to detect association than simple methods, such as the Bonferroni correction (or an equivalent such as permutation testing that takes into account correlation of the SNPs), where there are multiple SNPs associated with disease [19]. We therefore tested the hypothesis that subsets of the SNPs we have assessed are associated with breast cancer. Using the AML method, we found evidence for an overall association between common genetic variation in 120 candidate genes and breast cancer. In particular, we found some evidence of an association with SNPs in genes involved in cell-cycle control and steroid hormone metabolism. We also found evidence for population stratification in our study using data from 280 genomic control SNPs and corrected for this in all the analyses. However, the estimate of the inflation factor based on 280 SNPs is imprecise. We therefore carried out a sensitivity analysis using more extreme values of the inflation factor based on its variance. Under all but the most extreme assumptions (i.e., the upper 95% confidence limit of the inflation factor estimate) the global test of association remained significant. We therefore conclude that some proportion of the variants we have investigated are likely to be associated with breast cancer. In support of this conclusion, it is notable that stronger evidence of association has emerged for two of the SNPs we analysed, through collaborative analyses by the Breast Cancer Association Consortium [23]. One of these, in *CASP8,* was originally identified in another study but also showed evidence in our study [24]. The other, in *TGFB1,* was originally identified in a subset of cases and controls from our study [25].

Thus, our data provide further evidence for the existence of common low penetrance variants. The most efficient way to identify such variants is not clear, and considerable research funding and effort is currently being focussed on an empirical genome-wide approach rather than the candidate gene approach that has generally been the norm. The relative merits of the two approaches have not yet been defined, but our data suggest that the candidate gene approach may still be useful. However, our results also highlight the fact that alleles with modest effects, i.e., those conferring relative risks of >1.5, are likely to be the exception, and multicentre collaborations will be needed to generate adequate sample sizes.

## Materials and Methods

### Study participants.

Patients were drawn from Studies of Epidemiology and Risk factors in Cancer Heredity (SEARCH), an ongoing population-based study, with individuals ascertained through the East Anglian Cancer Registry. All patients diagnosed with invasive breast cancer below age 55 years since 1991 and still alive in 1996 (prevalent cases, median age 48 years), together with all those diagnosed below age 70 years between 1996 and the present (incident cases, median age 54 years) are eligible to take part. As of 1 August 2005 there have been 12,767 eligible patients. Of these, 2,284 were not contacted because their general practitioner did not respond or thought that it would be inappropriate to contact the patient. Of the 10,583 patients who were contacted, 67% have returned a questionnaire, and 64% provided a blood sample for DNA analysis. Eligible patients who did not take part in the study were similar to participants except, as might be expected, the proportion of clinical stage III/IV cases was somewhat higher in nonparticipants (10% versus 5%). Female controls were randomly selected from the Norfolk component of the European Prospective Investigation of Cancer (EPIC). EPIC is a prospective study of diet and cancer being carried out in nine European countries. The EPIC–Norfolk cohort comprises 25,000 individuals resident in Norfolk, East Anglia—the same region from which the cases have been recruited. Controls are not matched to cases, but are broadly similar in age (42–81 years). The ethnic background of both cases and controls as reported on the questionnaires is similar, with >98% being white. The study is approved by the Eastern Region Multicentre Research Ethics Committee, and all patients gave written informed consent.

The total number of cases used in genetic analyses was 4,473, of whom 27% are prevalent cases. The samples were split into two sets in order to save DNA and reduced genotyping costs: the first set (*n* = 2,270 cases and 2,280 controls) was genotyped for all SNPs, and the second set (*n* = 2,203 cases and 2,280 controls) were then tested for those SNPs that showed marginally significant associations in set 1 (*p*-heterogeneity or *p*-trend < 0.1). Two SNPs were genotyped in stage 2 as a result of a multimarker haplotype association (see below). This staged approach substantially reduces genotyping costs without significantly affecting statistical power. Cases were randomly selected for set 1 from the first 3,500 recruited, with set 2 comprising the remainder of these plus the next 974 incident cases recruited. As the prevalent cases were recruited first, the proportion of prevalent cases was somewhat higher in set 1 than set 2 (33% versus 20%). Median age at diagnosis is similar in both sets (51 and 52 years old, respectively). There was no significant difference in the morphology, histopathological grade, or clinical stage of the cases by set or by prevalent/incident status.

### Candidate gene and SNP selection.

We selected genes that encode proteins in cellular pathways that are likely to be involved in breast carcinogenesis. The major pathways we studied were steroid hormone metabolism and signalling, double strand break DNA repair, oxidative damage repair, epigenetic modifiers, and cell-cycle control. We also tested genes in the 17q21 region commonly amplified in breast tumours, several genes that have been found to be important in a variety of animal models of cancer, and some carcinogen metabolism genes. For some pathways, only a small subset of genes was selected for study. For the purpose of subgroup analysis, SNPs in these genes were categorised as “other” together with the SNPs in carcinogen metabolism genes. The genes and the number of SNPs assayed for each are shown in Table S1. The principal hypothesis underlying our approach is that there are one or more common SNPs in the genes of interest that are associated with an altered risk of breast cancer. We therefore aimed to identify a set of tagging SNPs (tSNPs) that efficiently tags all the known common variants (MAF > 0.05) and is likely to tag most of the unknown common variants. We used data from the International HapMap project (http://www.hapmap.org) or resequencing data from the National Institute of Environmental Health Sciences Environmental Genome Project (EGP) (http://www.niehs.nih.gov/envgenom/home.htm). The details of the methodology for tag SNP selection varied over time, but broadly speaking we have aimed to define a set of tagging SNPs such that all known common variants are correlated with a tSNP with *r* ^2^ of >0.8. Some SNPs are poorly correlated with other single SNPs but may be efficiently tagged by a haplotype defined by multiple SNPs, thus reducing the number of tagging SNPs needed [26]. As an alternative, therefore, we aimed for the correlation between each SNP and a haplotype of tagging SNPs to be at >0.8. For some genes, little information on the occurrence of common variants was available at the time the gene was studied, and the SNPs were selected for analysis based on predicted functional effects; Table S1 indicates which genes have been comprehensively tagged. We also obtained genotype data for our cases and controls for 280 randomly selected, unlinked SNPs, which were genotyped as part of an ongoing genome-wide association study (see Table S2). Data on these SNPs were used to adjust for population stratification using the genomic control method.

### Genotyping methods.

We genotyped all samples using the ABI PRISM 7900 sequence detection system or “Taqman” (Applied Biosystems, http://www.appliedbiosystems.com). Genomic DNA for set 1 samples was whole genome amplified by primer extension preamplification (PEP, protocol available on request). Genotype calling between PEP-amplified DNA and native genomic DNA was compared for eight Taqman assays, and the concordance was 100%. We carried out PCR on 10 ng of whole-genome amplified genomic DNA for set 1 and native genomic DNA for set 2 using TaqMan universal PCR master mix (Applied Biosystems), forward and reverse primers, and FAM- and VIC- labelled probes designed by Applied Biosystems (ABI Assay-by-Designs) in a 5-μl reaction. We read the completed PCRs on an ABI PRISM 7900 Sequence Detector in end-point mode using the Allelic Discrimination Sequence Detector software (Applied Biosystems). Cases and controls were arrayed together in 12 384-well plates, and a 13th plate contained eight duplicate samples from each of the 12 plates to ensure a good quality of genotyping. Each 384-well plate included two nontemplate controls. Concordance for duplicate samples was >98% for all assays. Failed genotypes were not repeated (the rate for failed genotypes did not exceed 8.3% for any of the SNPs under study). Genomic control SNPs were genotyped by Perlegen Sciences (http://www.perlegen.com) using an oligonucleotide array methodology.

### Statistical methods.

Association between disease and genotype for each SNP was assessed using two tests, the one-degree of freedom Cochran–Armitage trend test and the general two-degrees of freedom χ^2^ test (heterogeneity test). Results for all tests were summarised in Q–Q plots, in which the ordered test statistics are plotted against the expected statistic given the rank.

To assess the overall evidence for an excess of associations, we applied two approaches, the AML and RTP methods, which are described in detail elsewhere [17,19]. In brief, the AML method formulates the alternative hypothesis in terms of the probability that a given SNP is associated with disease (α) and a measure effect size. When a SNP is associated with disease, the calculated χ^2^ statistic will be distributed, asymptotically, as a noncentral χ^2^ distribution with the usual degrees of freedom and a noncentrality parameter η. The noncentrality parameter is a measure of the size of effect of the SNP, is dependent on sample size, and is closely related to the contribution of the SNP to the genetic variance of the trait. If η is assumed to be the same for each associated SNP, then both α and η can be estimated by maximum likelihood, and a test of the null hypothesis can then be derived as a likelihood ratio test. Where (as is the case here) some SNPs are correlated, the full likelihood is no longer straightforward, but pseudo-maximum likelihood estimates can still be generated by the same procedure, as if the SNPs were independent. Statistical significance can then be determined by simulation. The AML method was applied to both the trend and heterogeneity tests. The RTP is simply the product of the *K* (arbitrary) most significant *p*-values from *L* hypothesis tests [17]. A limitation of the RTP is the need to select a truncation point. While this may be straightforward in the context of a genome-wide study where it reduces the exploratory hypotheses to a defined candidate set, it is rather arbitrary in the context of candidate gene studies. For the purpose of these analyses we chose *K* = 5. We adjusted all analyses for cryptic population stratification using the method described by Devlin and Roeder [27]. An inflation factor (λ) was estimated from the mean of the X^2^_trend_ statistics generated on 280 unlinked, randomly selected SNPs typed on a subset of 4,037 cases and 4,012 controls as part of a separate genome-wide association study. A list of these SNPs is provided in the supplementary material. The average call rate was 99.0% in cases and 98.9% in controls. The inflation of the test statistic, adjusted for sample size, was estimated to be 1.15 (95% CI 0.94–1.36) for the trend test and 1.05 (95% CI 0.92–1.19) for the heterogeneity test.

Association tests for individual SNPs will not be independent if the markers are in linkage disequilibrium, and the application of both methods needs to allow for the correlation structure of the data. Simulations, based on permuting case-control status whilst retaining the correlation structure among makers, provide a robust approach for obtaining significance levels for these global tests. However, permutation testing is complicated by the use of a staged study design where only those SNPs significant in the first stage data are genotyped for the complete set of cases and controls. We allowed for this using the method proposed by Dudbridge [28] in which a subset of the first stage date is used as the simulated first stage data, selecting markers on the basis of that subset, and using the remainder of the first stage as the simulated second stage.

## Supporting Information

Table S1 Genes Investigated as Candidates for Breast Cancer Susceptibility by Pathway (137 KB DOC)

Table S2 Control and Case Genotype Frequencies for 280 Genomic Control SNPs (521 KB DOC)

Table S3 Genotype Frequencies in Controls and Cases for 710 Candidate Gene SNPs (1.0 MB DOC)

## Abbreviations

AML: admixture maximum likelihood
MAF: minor allele frequency
RTP: rank truncated product
SNP: single nucleotide polymorphism
