# Supplementary material for: Association between Common Variation in 120 Candidate Genes and Breast Cancer Risk
Source: PLoS Genet. 2007 Mar 16;3(3):e42. doi: 10.1371/journal.pgen.0030042 (PMC1828694; doi:10.1371/journal.pgen.0030042)
Supplement: Supplementary file 1 [file pgen.0030042.st001.doc]

**Table S**1: Genes investigated as candidates for breast cancer susceptibility by pathway

| Gene | SNPs assayed | Comprehensive tagging |  | Gene | SNPs assayed | Comprehensive tagging |
| --- | --- | --- | --- | --- | --- | --- |
| 17q21 amplicon |  |  |  | Epigenetic modifiers |  |  |
| C17ORF37 | 1 | Yes |  | BAT8 | 2 | Yes |
| CAB2 | 3 | Yes |  | CBP | 6 | Yes |
| CRK7 | 2 | Yes |  | DNMT1 | 2 | Yes |
| ERBB2 | 7 | Yes |  | DNMT3A | 14 | Yes |
| GRB7 | 2 | Yes |  | DNMT3B | 9 | Yes |
| NEUROD2 | 1 | Yes |  | EHMT1 | 6 | Yes |
| PPARBP | 3 | Yes |  | HDAC2 | 6 | Yes |
| PPP1R1B | 2 | Yes |  | HDAC5 | 3 | Yes |
| STARD3 | 1 | Yes |  | MBD2 | 4 | Yes |
| ZNFN1A3 | 2 | Yes |  | MTHFR | 1 | No |
| Cell cycle control |  |  |  | P300 | 4 | No |
| ATR | 6 | Yes |  | RIZ1 | 8 | Yes |
| CCND1 | 7 | Yes |  | SETDB1 | 2 | Yes |
| CCND2 | 14 | Yes |  | Animal models |  |  |
| CCND3 | 7 | Yes |  | ADAM17 | 3 | No |
| CCNE1 | 4 | Yes |  | CSTF1 | 2 | No |
| CDK2 | 2 | Yes |  | FASL | 4 | No |
| CDK4 | 1 | Yes |  | ICAP1B | 1 | No |
| CDK6 | 13 | Yes |  | MCS5A6 | 5 | Yes |
| CDKN1A | 9 | Yes |  | P5 | 1 | No |
| CDKN1B | 8 | Yes |  | PCNXL3 | 1 | No |
| CDKN2A | 13 | Yes |  | PPP1CB | 1 | No |
| CDKN2B | 3 | Yes |  | PTPRJ | 5 | Yes |
| CDKN2C | 2 | Yes |  | RELA | 5 | Yes |
| CDKN2D | 1 | Yes |  | STK15 | 4 | No |
| E2F5 | 1 | No |  | TIEG2 | 2 | No |
| FBXW7 | 1 | No |  | Other |  |  |
| RB1 | 13 | Yes |  | ADH1A | 2 | Yes |
| SIPA1 | 3 | No |  | ADH1B | 3 | Yes |
| DNA repair |  |  |  | ADH1C | 3 | Yes |
| ATM | 10 | Yes |  | CASP8 | 3 | No |
| BARD1 | 11 | Yes |  | GSTM1D | 1 | No |
| BRCA1 | 5 | Yes |  | ICAM5 | 1 | No |
| BRCA2 | 16 | Yes |  | MMP13 | 1 | No |
| BRIP1 | 13 | Yes |  | MMP2 | 1 | No |
| CHEK1 | 6 | Yes |  | MMP8 | 9 | No |
| CHEK2 | 10 | Yes |  | MUS81 | 1 | No |
| EMSY | 8 | Yes |  | NR1I2 | 8 | Yes |
| LIG4 | 7 | Yes |  | NUMA1 | 1 | No |
| MDM2 | 6 | Yes |  | PGDS | 1 | Yes |
| MRE11A | 7 | Yes |  | PPP1R2P1 | 1 | Yes |
| NBS1 | 8 | Yes |  | PTGDS | 3 | Yes |
| RAD50 | 5 | Yes |  | PTGES | 9 | Yes |
| RAD51 | 6 | Yes |  | PTGIS | 15 | Yes |
| RAD51C | 5 | Yes |  | PTGS1 | 5 | Yes |
| RAD52 | 12 | Yes |  | PTGS2 | 4 | Yes |
| RAD54 | 7 | Yes |  | TBXAS1 | 25 | Yes |
| TP53 | 6 | Yes |  | Oxidative damage repair |  |  |
| XRCC2 | 9 |  |  | ABL1 | 5 | Yes |
| XRCC3 | 7 |  |  | CAT | 5 | Yes |
| Growth factors |  |  |  | GPX1 | 1 | Yes |
| EGF | 1 | No |  | GPX4 | 5 | Yes |
| IGF1 | 10 | Yes |  | GSR | 10 | Yes |
| IGF1R | 13 | Yes |  | SOD1 | 3 | Yes |
| IGF2 | 6 | Yes |  | SOD2 | 3 | Yes |
| IGFBP3 | 4 | Yes |  | TXN | 15 | Yes |
| IL10 | 4 | Yes |  | TXN2 | 3 | Yes |
| TGFB1 | 1 | Yes |  | TXNRD1 | 4 | Yes |
| VEGF | 4 | Yes |  | TXNRD2 | 11 | Yes |
|  |  |  |  | Steroid hormone metabolism and signalling | | |
|  |  |  |  | COMT | 2 | No |
|  |  |  |  | CYP19 | 2 | No |
|  |  |  |  | ESR1 | 69 | No |
|  |  |  |  | ESR2 | 2 | No |
|  |  |  |  | PGR | 10 | Yes |
|  |  |  |  | SHBG | 7 | Yes |
|  |  |  |  | STE | 9 | Yes |
|  |  |  |  | STS | 3 | Yes |
