# Supplementary material for: Association between Common Variation in 120 Candidate Genes and Breast Cancer Risk
Source: PLoS Genet. 2007 Mar 16;3(3):e42. doi: 10.1371/journal.pgen.0030042 (PMC1828694; doi:10.1371/journal.pgen.0030042)
Supplement: Supplementary file 2 [file pgen.0030042.st002.doc]

**Table S2: Control and case genotype frequencies for 280 genomic cont**rol SNPs

|  |  | Controls |  |  | Cases |  |
| --- | --- | --- | --- | --- | --- | --- |
| SNP | AA | Aa | aa | AA | Aa | aa |
| rs9525158 | 1170 | 1934 | 888 | 1110 | 2010 | 890 |
| rs9332472 | 1245 | 1965 | 794 | 1228 | 2018 | 786 |
| rs9332471 | 3524 | 446 | 16 | 3510 | 479 | 19 |
| rs9332470 | 2389 | 1383 | 205 | 2419 | 1352 | 239 |
| rs9332469 | 1069 | 2007 | 928 | 1027 | 2053 | 950 |
| rs9332468 | 2058 | 1617 | 331 | 2054 | 1669 | 310 |
| rs9332467 | 1577 | 1852 | 552 | 1576 | 1847 | 590 |
| rs9332466 | 2082 | 1562 | 300 | 2234 | 1468 | 260 |
| rs9332465 | 1068 | 1967 | 966 | 1092 | 1985 | 951 |
| rs9332464 | 2490 | 1332 | 177 | 2528 | 1315 | 185 |
| rs9332463 | 1565 | 1875 | 559 | 1506 | 1907 | 603 |
| rs9332462 | 1253 | 1964 | 765 | 1254 | 1957 | 802 |
| rs9332461 | 1512 | 1910 | 580 | 1501 | 1884 | 645 |
| rs9332459 | 1435 | 1855 | 607 | 1414 | 1874 | 654 |
| rs9332457 | 1233 | 1976 | 777 | 1313 | 1946 | 760 |
| rs9332456 | 1062 | 2039 | 906 | 1114 | 2012 | 904 |
| rs9332455 | 1781 | 1766 | 452 | 1790 | 1751 | 483 |
| rs9332454 | 1332 | 1979 | 694 | 1297 | 1962 | 776 |
| rs9332453 | 1546 | 1861 | 592 | 1488 | 1902 | 636 |
| rs9332452 | 1475 | 1882 | 638 | 1497 | 1923 | 606 |
| rs9332450 | 1849 | 1720 | 414 | 1902 | 1720 | 394 |
| rs9332449 | 1712 | 1801 | 463 | 1725 | 1802 | 485 |
| rs9332448 | 1155 | 1969 | 882 | 1105 | 2069 | 861 |
| rs9332447 | 1227 | 2013 | 769 | 1327 | 1940 | 762 |
| rs9332446 | 1063 | 1963 | 920 | 1055 | 1972 | 943 |
| rs9332445 | 1453 | 1920 | 629 | 1419 | 1972 | 637 |
| rs9332444 | 1938 | 1673 | 355 | 1960 | 1665 | 382 |
| rs9332443 | 3082 | 858 | 67 | 3052 | 909 | 67 |
| rs9332442 | 1356 | 1755 | 657 | 1430 | 1728 | 668 |
| rs9332441 | 2275 | 1502 | 230 | 2290 | 1482 | 255 |
| rs9332437 | 2016 | 1666 | 323 | 2039 | 1626 | 365 |
| rs9332436 | 2154 | 1567 | 281 | 2182 | 1551 | 289 |
| rs9332435 | 1986 | 1682 | 335 | 2005 | 1652 | 360 |
| rs9332434 | 1109 | 1999 | 892 | 1070 | 2001 | 942 |
| rs9332433 | 1604 | 1798 | 588 | 1614 | 1851 | 541 |
| rs9332432 | 1404 | 1895 | 697 | 1407 | 1930 | 671 |
| rs9332431 | 1202 | 1910 | 876 | 1197 | 1982 | 836 |
| rs9332430 | 1568 | 1783 | 630 | 1534 | 1833 | 639 |
| rs9332429 | 1822 | 1697 | 407 | 1859 | 1695 | 403 |
| rs9332428 | 1359 | 1951 | 681 | 1437 | 1900 | 678 |
| rs9332427 | 1464 | 1902 | 630 | 1433 | 1923 | 657 |
| rs9332426 | 1618 | 1812 | 557 | 1588 | 1884 | 546 |
| rs9332425 | 1509 | 1910 | 576 | 1523 | 1868 | 636 |
| rs9332424 | 1174 | 1964 | 863 | 1135 | 2036 | 850 |
| rs9332423 | 1116 | 1956 | 918 | 1124 | 1959 | 929 |
| rs9332422 | 1259 | 1949 | 790 | 1209 | 1977 | 832 |
| rs9332421 | 1215 | 1963 | 813 | 1246 | 1995 | 773 |
| rs9332420 | 2006 | 1661 | 328 | 1952 | 1696 | 374 |
| rs9332419 | 1444 | 1873 | 662 | 1439 | 1944 | 632 |
| rs9332418 | 2552 | 1293 | 153 | 2513 | 1338 | 178 |
| rs9332417 | 1337 | 2001 | 665 | 1357 | 1952 | 717 |
| rs9332416 | 1685 | 1806 | 499 | 1735 | 1808 | 476 |
| rs9332415 | 2011 | 1598 | 295 | 1973 | 1647 | 322 |
| rs9332414 | 1678 | 1827 | 498 | 1747 | 1801 | 474 |
| rs9332413 | 1380 | 1933 | 691 | 1291 | 2019 | 713 |
| rs9332412 | 1260 | 1924 | 781 | 1236 | 1989 | 773 |
| rs9332411 | 1240 | 1940 | 745 | 1301 | 1899 | 753 |
| rs9332410 | 1616 | 1846 | 532 | 1603 | 1864 | 547 |
| rs9332409 | 1520 | 1877 | 587 | 1533 | 1900 | 579 |
| rs9332408 | 1194 | 1914 | 866 | 1081 | 1985 | 930 |
| rs9332407 | 1135 | 1980 | 891 | 1154 | 1989 | 888 |
| rs9332406 | 1537 | 1890 | 571 | 1603 | 1856 | 571 |
| rs9332405 | 1977 | 1677 | 344 | 1951 | 1670 | 402 |
| rs9332404 | 2180 | 1542 | 278 | 2168 | 1555 | 302 |
| rs9332403 | 1033 | 2004 | 954 | 1087 | 2024 | 918 |
| rs9332402 | 1244 | 1941 | 782 | 1277 | 1937 | 770 |
| rs9332401 | 2586 | 1249 | 165 | 2646 | 1241 | 140 |
| rs9332399 | 1862 | 1697 | 403 | 1941 | 1635 | 404 |
| rs9332393 | 2451 | 1320 | 180 | 2470 | 1316 | 191 |
| rs9332392 | 1950 | 1646 | 370 | 1900 | 1701 | 392 |
| rs9332390 | 1096 | 1938 | 960 | 1096 | 2027 | 901 |
| rs9332389 | 1480 | 1881 | 641 | 1455 | 1949 | 615 |
| rs9332387 | 2198 | 1537 | 266 | 2186 | 1543 | 293 |
| rs9332386 | 2427 | 1308 | 224 | 2464 | 1329 | 203 |
| rs9328058 | 1544 | 1860 | 575 | 1558 | 1856 | 594 |
| rs9324014 | 1581 | 1885 | 520 | 1630 | 1844 | 540 |
| rs9323327 | 1334 | 1933 | 732 | 1381 | 1938 | 706 |
| rs9322330 | 3917 | 88 | 1 | 3931 | 83 | 2 |
| rs9318026 | 3087 | 847 | 66 | 3128 | 795 | 96 |
| rs9315543 | 1103 | 2009 | 886 | 1049 | 1968 | 1000 |
| rs9313204 | 1369 | 1951 | 684 | 1346 | 2028 | 655 |
| rs9310422 | 1319 | 1906 | 768 | 1264 | 1960 | 799 |
| rs9306906 | 3225 | 741 | 30 | 3234 | 738 | 51 |
| rs9306469 | 2376 | 1389 | 226 | 2416 | 1385 | 222 |
| rs9303777 | 998 | 2015 | 992 | 1061 | 2044 | 925 |
| rs9303397 | 1426 | 1896 | 664 | 1464 | 1905 | 656 |
| rs9298877 | 1059 | 1983 | 951 | 1041 | 1990 | 987 |
| rs9298162 | 1024 | 1946 | 970 | 1048 | 1983 | 948 |
| rs9294997 | 2946 | 976 | 83 | 2952 | 974 | 106 |
| rs9291659 | 1430 | 1881 | 693 | 1428 | 1923 | 673 |
| rs9290474 | 989 | 2026 | 932 | 1001 | 2056 | 895 |
| rs9288801 | 1875 | 1715 | 411 | 1838 | 1755 | 428 |
| rs9288593 | 1939 | 1676 | 389 | 1924 | 1707 | 400 |
| rs9285863 | 1803 | 1734 | 463 | 1762 | 1778 | 485 |
| rs9285235 | 1345 | 1883 | 700 | 1282 | 1911 | 680 |
| rs9284788 | 1144 | 1948 | 878 | 1141 | 1996 | 859 |
| rs904462 | 3505 | 483 | 16 | 3521 | 482 | 19 |
| rs883399 | 1386 | 1974 | 645 | 1460 | 1870 | 695 |
| rs854809 | 1847 | 1774 | 375 | 1850 | 1728 | 443 |
| rs850157 | 2035 | 1624 | 322 | 2070 | 1609 | 331 |
| rs8180040 | 1396 | 1935 | 653 | 1432 | 1960 | 616 |
| rs8106462 | 1373 | 1931 | 680 | 1387 | 1945 | 681 |
| rs8089528 | 1756 | 1791 | 450 | 1744 | 1824 | 455 |
| rs8071941 | 1149 | 1460 | 349 | 1410 | 1724 | 460 |
| rs8062983 | 1574 | 1868 | 544 | 1583 | 1878 | 559 |
| rs8046880 | 3015 | 920 | 68 | 3086 | 871 | 73 |
| rs8041414 | 2770 | 1113 | 115 | 2741 | 1151 | 129 |
| rs8023865 | 3066 | 861 | 56 | 3090 | 858 | 70 |
| rs8013489 | 1595 | 1853 | 542 | 1616 | 1894 | 509 |
| rs8008169 | 1347 | 1986 | 665 | 1369 | 1954 | 697 |
| rs7990319 | 1230 | 1963 | 800 | 1288 | 2001 | 727 |
| rs7960855 | 1258 | 1897 | 731 | 1226 | 1946 | 760 |
| rs7916571 | 1736 | 1745 | 524 | 1731 | 1797 | 498 |
| rs7818744 | 1538 | 1863 | 580 | 1567 | 1861 | 577 |
| rs780846 | 2310 | 1470 | 226 | 2243 | 1536 | 248 |
| rs775219 | 2125 | 1565 | 311 | 2023 | 1665 | 331 |
| rs7713108 | 2657 | 1193 | 151 | 2642 | 1226 | 158 |
| rs7703210 | 1108 | 1966 | 827 | 1076 | 2020 | 818 |
| rs7697319 | 1740 | 1832 | 410 | 1736 | 1820 | 452 |
| rs7690426 | 1098 | 1997 | 842 | 1141 | 1940 | 876 |
| rs7664025 | 1835 | 1763 | 403 | 1874 | 1721 | 432 |
| rs7659335 | 1283 | 1884 | 741 | 1355 | 1870 | 736 |
| rs7645596 | 2122 | 1578 | 292 | 2110 | 1563 | 334 |
| rs7638306 | 1749 | 1722 | 461 | 1736 | 1771 | 445 |
| rs7612632 | 1771 | 1723 | 489 | 1759 | 1808 | 450 |
| rs7584345 | 1974 | 1603 | 342 | 1920 | 1594 | 372 |
| rs7581465 | 1192 | 1905 | 889 | 1143 | 1952 | 913 |
| rs7517189 | 3312 | 633 | 32 | 3293 | 679 | 45 |
| rs7338389 | 1791 | 1750 | 448 | 1647 | 1702 | 484 |
| rs7315267 | 1544 | 1791 | 480 | 1536 | 1783 | 530 |
| rs7304475 | 1453 | 1901 | 635 | 1403 | 1969 | 627 |
| rs7296765 | 1158 | 1959 | 870 | 1148 | 1982 | 883 |
| rs7225855 | 1283 | 1979 | 715 | 1286 | 1985 | 746 |
| rs7168889 | 1118 | 2005 | 871 | 1162 | 1982 | 879 |
| rs7082609 | 1132 | 1940 | 931 | 1063 | 2026 | 934 |
| rs708255 | 1011 | 1940 | 954 | 1020 | 1977 | 926 |
| rs6987013 | 1500 | 1847 | 618 | 1446 | 1904 | 636 |
| rs6982484 | 2145 | 1509 | 270 | 2207 | 1513 | 257 |
| rs6901567 | 1510 | 1856 | 627 | 1556 | 1861 | 604 |
| rs687559 | 1060 | 1912 | 1033 | 970 | 2038 | 1017 |
| rs685912 | 2656 | 1212 | 140 | 2637 | 1236 | 155 |
| rs6855142 | 1892 | 1729 | 355 | 1955 | 1703 | 352 |
| rs6845147 | 1452 | 1896 | 630 | 1453 | 1859 | 687 |
| rs6841855 | 1127 | 2023 | 849 | 1169 | 2039 | 818 |
| rs6797692 | 2533 | 1315 | 160 | 2491 | 1352 | 181 |
| rs6766000 | 3016 | 887 | 83 | 2980 | 948 | 80 |
| rs6758386 | 1065 | 1902 | 991 | 1019 | 1974 | 1007 |
| rs6716388 | 1282 | 1941 | 784 | 1247 | 2059 | 725 |
| rs6692705 | 1387 | 1891 | 673 | 1458 | 1855 | 665 |
| rs6674638 | 2248 | 1493 | 260 | 2241 | 1498 | 280 |
| rs6660559 | 1021 | 2035 | 942 | 1044 | 2027 | 965 |
| rs6592508 | 1082 | 2024 | 888 | 1122 | 1999 | 900 |
| rs6564337 | 1341 | 1961 | 700 | 1425 | 1933 | 664 |
| rs6549011 | 1635 | 1733 | 424 | 1558 | 1853 | 453 |
| rs6538463 | 1442 | 1906 | 647 | 1400 | 1951 | 667 |
| rs6471542 | 1824 | 1758 | 415 | 1794 | 1816 | 405 |
| rs6470637 | 2971 | 964 | 73 | 2993 | 966 | 69 |
| rs6449586 | 1274 | 1915 | 798 | 1279 | 1902 | 837 |
| rs644016 | 1731 | 1760 | 466 | 1699 | 1789 | 480 |
| rs6439591 | 1285 | 1919 | 774 | 1319 | 1988 | 702 |
| rs6136556 | 1812 | 1734 | 447 | 1852 | 1716 | 446 |
| rs6042018 | 1616 | 1823 | 514 | 1646 | 1796 | 547 |
| rs6018600 | 1294 | 1974 | 700 | 1408 | 1899 | 696 |
| rs590991 | 1149 | 1963 | 885 | 1184 | 1974 | 864 |
| rs582883 | 1941 | 1695 | 370 | 1920 | 1718 | 389 |
| rs5754891 | 1091 | 2044 | 859 | 1055 | 2081 | 882 |
| rs5748736 | 1074 | 1958 | 958 | 1053 | 1983 | 978 |
| rs553696 | 1782 | 1725 | 472 | 1820 | 1735 | 458 |
| rs512398 | 1153 | 1973 | 856 | 1134 | 1992 | 891 |
| rs5019250 | 1185 | 2018 | 801 | 1164 | 1994 | 871 |
| rs4938659 | 1379 | 1943 | 678 | 1489 | 1885 | 652 |
| rs4933583 | 1980 | 1627 | 342 | 2023 | 1643 | 340 |
| rs4921056 | 2136 | 1564 | 307 | 2101 | 1626 | 302 |
| rs4879670 | 1363 | 1901 | 688 | 1363 | 1930 | 699 |
| rs4871877 | 1842 | 1679 | 459 | 1853 | 1744 | 410 |
| rs4847431 | 1483 | 1843 | 599 | 1463 | 1886 | 630 |
| rs4841224 | 1887 | 1721 | 395 | 1897 | 1759 | 369 |
| rs4810264 | 1741 | 1790 | 467 | 1693 | 1822 | 505 |
| rs4808631 | 1422 | 1928 | 646 | 1424 | 1923 | 672 |
| rs478112 | 1110 | 1972 | 919 | 1090 | 2001 | 933 |
| rs4766951 | 1066 | 1986 | 952 | 1048 | 2007 | 968 |
| rs4746308 | 3314 | 656 | 36 | 3341 | 657 | 29 |
| rs4734695 | 1601 | 1811 | 589 | 1593 | 1848 | 578 |
| rs4733308 | 1377 | 1892 | 736 | 1328 | 2015 | 687 |
| rs4725500 | 1079 | 1964 | 951 | 1127 | 2021 | 879 |
| rs4720913 | 1218 | 1976 | 794 | 1215 | 1929 | 863 |
| rs4712961 | 2085 | 1564 | 353 | 2096 | 1602 | 331 |
| rs4706947 | 1753 | 1789 | 456 | 1729 | 1843 | 454 |
| rs4673990 | 1155 | 2013 | 832 | 1182 | 1980 | 856 |
| rs4656256 | 2537 | 1270 | 179 | 2499 | 1313 | 194 |
| rs4637055 | 2275 | 1493 | 221 | 2264 | 1524 | 232 |
| rs451061 | 1497 | 1859 | 555 | 1507 | 1891 | 570 |
| rs4475815 | 1561 | 1865 | 568 | 1519 | 1900 | 604 |
| rs4466264 | 1683 | 1828 | 490 | 1663 | 1828 | 532 |
| rs4445211 | 1711 | 1777 | 512 | 1691 | 1842 | 490 |
| rs4424659 | 1202 | 1988 | 793 | 1174 | 1992 | 853 |
| rs4424334 | 1622 | 1850 | 523 | 1585 | 1878 | 557 |
| rs4382428 | 1307 | 1950 | 725 | 1253 | 1988 | 769 |
| rs4321804 | 1115 | 2012 | 880 | 1102 | 2047 | 888 |
| rs4318528 | 1051 | 1969 | 970 | 1090 | 1933 | 985 |
| rs425623 | 1364 | 1850 | 646 | 1303 | 1909 | 692 |
| rs4236492 | 1551 | 1612 | 337 | 1640 | 1636 | 336 |
| rs4233602 | 1359 | 1868 | 654 | 1433 | 1855 | 625 |
| rs4075276 | 2102 | 1624 | 281 | 2171 | 1558 | 301 |
| rs387816 | 1353 | 1953 | 692 | 1426 | 1940 | 658 |
| rs3821502 | 1405 | 1900 | 688 | 1424 | 1900 | 688 |
| rs3820667 | 1219 | 1969 | 818 | 1196 | 1979 | 846 |
| rs3815901 | 1257 | 1920 | 790 | 1296 | 1925 | 780 |
| rs3801303 | 1026 | 1989 | 919 | 1037 | 2030 | 935 |
| rs3796734 | 1278 | 1947 | 753 | 1314 | 1966 | 735 |
| rs3776168 | 1712 | 1813 | 480 | 1748 | 1788 | 493 |
| rs37545 | 1468 | 1896 | 630 | 1547 | 1880 | 598 |
| rs3752523 | 1275 | 1966 | 744 | 1260 | 1989 | 746 |
| rs350993 | 1851 | 1709 | 424 | 1812 | 1744 | 446 |
| rs331762 | 1210 | 2010 | 774 | 1219 | 1994 | 811 |
| rs313757 | 1213 | 1959 | 815 | 1223 | 1968 | 811 |
| rs31251 | 1345 | 1931 | 711 | 1350 | 1959 | 713 |
| rs309370 | 1843 | 1694 | 429 | 1797 | 1731 | 473 |
| rs2996245 | 2304 | 1435 | 255 | 2325 | 1462 | 232 |
| rs2975997 | 1705 | 1829 | 466 | 1663 | 1854 | 508 |
| rs2967532 | 1303 | 1684 | 721 | 1344 | 1818 | 659 |
| rs2962975 | 1952 | 1661 | 388 | 1901 | 1732 | 399 |
| rs291402 | 2185 | 1558 | 258 | 2317 | 1455 | 251 |
| rs2853362 | 1523 | 1879 | 591 | 1439 | 1911 | 672 |
| rs2836168 | 1168 | 1959 | 791 | 1166 | 1995 | 793 |
| rs2832691 | 1073 | 1878 | 602 | 1290 | 1845 | 641 |
| rs2826689 | 1630 | 1821 | 546 | 1588 | 1892 | 537 |
| rs2796188 | 1057 | 1967 | 962 | 1048 | 2013 | 959 |
| rs2794410 | 1759 | 1754 | 455 | 1739 | 1757 | 493 |
| rs2760101 | 1436 | 1905 | 536 | 1421 | 1914 | 599 |
| rs2733282 | 1099 | 2037 | 865 | 1071 | 2027 | 932 |
| rs2715368 | 1580 | 1829 | 583 | 1635 | 1853 | 529 |
| rs2702636 | 1980 | 1575 | 366 | 1908 | 1696 | 366 |
| rs2572357 | 2040 | 1642 | 317 | 1971 | 1686 | 357 |
| rs2567661 | 2934 | 983 | 79 | 2976 | 958 | 87 |
| rs2518128 | 1522 | 1589 | 365 | 1540 | 1692 | 380 |
| rs2515366 | 1321 | 1933 | 736 | 1338 | 1954 | 732 |
| rs2513993 | 2204 | 1530 | 272 | 2152 | 1592 | 284 |
| rs2502521 | 1831 | 1713 | 447 | 1799 | 1743 | 460 |
| rs2475803 | 1422 | 1931 | 644 | 1481 | 1884 | 659 |
| rs2444975 | 1058 | 1919 | 1012 | 1049 | 1963 | 1006 |
| rs2423422 | 2309 | 1425 | 259 | 2307 | 1479 | 230 |
| rs2303703 | 2042 | 1609 | 345 | 1991 | 1688 | 346 |
| rs2302565 | 1774 | 1810 | 423 | 1826 | 1755 | 448 |
| rs2297596 | 1368 | 1953 | 660 | 1409 | 1929 | 680 |
| rs2295706 | 2823 | 1091 | 88 | 2783 | 1132 | 106 |
| rs2294771 | 1991 | 1630 | 337 | 1980 | 1671 | 326 |
| rs2290144 | 2280 | 1280 | 172 | 2348 | 1256 | 167 |
| rs2287403 | 1129 | 1947 | 893 | 1134 | 1985 | 894 |
| rs2270474 | 1016 | 2039 | 928 | 1057 | 2017 | 953 |
| rs2255663 | 1985 | 1622 | 373 | 2006 | 1660 | 323 |
| rs2254826 | 1190 | 1924 | 864 | 1130 | 1965 | 891 |
| rs225277 | 1693 | 1810 | 499 | 1663 | 1851 | 515 |
| rs2235927 | 2021 | 1628 | 342 | 2000 | 1627 | 376 |
| rs2234246 | 1004 | 2002 | 975 | 1001 | 1972 | 1028 |
| rs2230601 | 1184 | 2022 | 792 | 1164 | 2004 | 859 |
| rs217378 | 1250 | 2000 | 757 | 1253 | 2030 | 748 |
| rs212968 | 1714 | 1782 | 487 | 1778 | 1747 | 486 |
| rs212772 | 1136 | 1974 | 890 | 1222 | 1967 | 839 |
| rs208507 | 3057 | 884 | 48 | 3061 | 886 | 61 |
| rs1845789 | 1983 | 1632 | 384 | 1968 | 1708 | 339 |
| rs17712237 | 1217 | 1955 | 812 | 1160 | 1982 | 873 |
| rs17662685 | 2183 | 1527 | 289 | 2209 | 1549 | 263 |
| rs17187953 | 1624 | 1723 | 416 | 1593 | 1761 | 415 |
| rs1714509 | 1086 | 1954 | 869 | 1091 | 1976 | 882 |
| rs17058987 | 1432 | 1912 | 659 | 1446 | 1979 | 608 |
| rs1629422 | 1932 | 1637 | 352 | 1852 | 1719 | 385 |
| rs1620326 | 1072 | 2037 | 872 | 1066 | 2019 | 930 |
| rs1582091 | 1082 | 1978 | 932 | 1105 | 1947 | 967 |
| rs156093 | 1724 | 1765 | 504 | 1696 | 1870 | 450 |
| rs1549957 | 1386 | 1948 | 660 | 1340 | 1966 | 710 |
| rs1480106 | 2411 | 1406 | 186 | 2437 | 1372 | 217 |
| rs1400882 | 1388 | 1889 | 716 | 1385 | 1970 | 677 |
| rs1268169 | 1698 | 1793 | 506 | 1703 | 1825 | 489 |
| rs1263605 | 1640 | 1830 | 502 | 1637 | 1839 | 530 |
| rs12533584 | 1900 | 1711 | 395 | 1917 | 1714 | 398 |
| rs11693885 | 1239 | 1987 | 776 | 1278 | 1938 | 804 |
| rs10841006 | 978 | 1994 | 928 | 1017 | 2002 | 943 |
| rs10489218 | 913 | 1873 | 672 | 939 | 1848 | 705 |
| rs10068004 | 1643 | 1763 | 517 | 1670 | 1860 | 438 |
