# Supplementary material for: Association between Common Variation in 120 Candidate Genes and Breast Cancer Risk
Source: PLoS Genet. 2007 Mar 16;3(3):e42. doi: 10.1371/journal.pgen.0030042 (PMC1828694; doi:10.1371/journal.pgen.0030042)
Supplement: Supplementary file 3 [file pgen.0030042.st003.doc]

**Table S**3: Genotype frequencies in controls and cases for 710 candidate gene SNPs

| Gene | dBSNP ref * |  | Controls |  |  | Cases |  |
| --- | --- | --- | --- | --- | --- | --- | --- |
|  |  | AA | Aa | aa | AA | Aa | aa |
| ABL1 | rs2480372 | 1672 | 559 | 47 | 1584 | 563 | 47 |
| ABL1 | rs2855158 | 1174 | 921 | 180 | 1148 | 887 | 156 |
| ABL1 | rs7457 | 1914 | 288 | 12 | 1746 | 281 | 10 |
| ABL1 | rs2070997 | 1820 | 432 | 26 | 1746 | 421 | 27 |
| ABL1 | rs2791743 | 1334 | 813 | 131 | 1266 | 811 | 117 |
| ADAM17 | S747L | 2117 | 77 | 1 | 1969 | 66 | 1 |
| ADAM17 | 2714a>g | 777 | 1068 | 326 | 754 | 937 | 354 |
| ADAM17 | -144a>g | 1016 | 958 | 203 | 931 | 875 | 222 |
| ADH1A | rs1229976 | 1361 | 792 | 111 | 1270 | 783 | 119 |
| ADH1A | rs13123099 | 900 | 1018 | 288 | 830 | 975 | 299 |
| ADH1B | rs1229984 | 2045 | 129 | 3 | 1894 | 106 | 6 |
| ADH1B | rs17033 | 1893 | 358 | 20 | 1829 | 337 | 14 |
| ADH1B | rs1042026 | 2159 | 1900 | 371 | 2159 | 1602 | 365 |
| ADH1C | rs1614972 | 1088 | 963 | 215 | 1097 | 900 | 185 |
| ADH1C | rs1789920 | 1068 | 931 | 190 | 959 | 863 | 205 |
| ADH1C | rs698 | 1627 | 2127 | 699 | 1473 | 1995 | 743 |
| ATM | rs1800054 | 4423 | 126 | 0 | 4219 | 138 | 2 |
| ATM | rs1800889 | 2043 | 214 | 13 | 1990 | 183 | 9 |
| ATM | rs665293 | 1052 | 1000 | 222 | 990 | 971 | 227 |
| ATM | rs2234996 | 2103 | 169 | 6 | 1992 | 183 | 5 |
| ATM | rs582157 | 712 | 1110 | 405 | 687 | 1037 | 426 |
| ATM | rs599164 | 727 | 1147 | 398 | 712 | 1059 | 409 |
| ATM | rs4987876 | 1881 | 378 | 18 | 1808 | 364 | 21 |
| ATM | rs189037 | 707 | 1146 | 420 | 693 | 1070 | 427 |
| ATM | rs3092991 | 3280 | 1193 | 82 | 3224 | 1080 | 74 |
| ATM | rs611018 | 1945 | 310 | 22 | 1874 | 298 | 14 |
| ATR | rs9856772 | 1768 | 461 | 29 | 1746 | 403 | 26 |
| ATR | rs2229032 | 1594 | 608 | 69 | 1528 | 595 | 58 |
| ATR | rs11920625 | 1858 | 389 | 17 | 1777 | 370 | 20 |
| ATR | rs6805118 | 1875 | 375 | 24 | 1766 | 397 | 16 |
| ATR | rs10804682 | 1471 | 716 | 90 | 1402 | 704 | 78 |
| ATR | rs2227928 | 772 | 1093 | 401 | 770 | 1054 | 352 |
| ATR | rs13091637 | 1463 | 711 | 98 | 1369 | 711 | 107 |
| ATR | rs1802904 | 1659 | 553 | 54 | 1630 | 509 | 41 |
| ATR | rs6440085 | 846 | 1057 | 364 | 764 | 1041 | 366 |
| BARD1 | rs6759222 | 2639 | 1609 | 258 | 2613 | 1482 | 230 |
| BARD1 | rs3768704 | 3516 | 927 | 75 | 3422 | 832 | 70 |
| BARD1 | rs2075623 | 864 | 1047 | 354 | 819 | 1019 | 337 |
| BARD1 | rs6435862 | 2397 | 1789 | 349 | 2193 | 1836 | 324 |
| BARD1 | rs6717301 | 3279 | 1123 | 97 | 3168 | 1064 | 100 |
| BARD1 | rs7591615 | 2546 | 1674 | 261 | 2507 | 1555 | 254 |
| BARD1 | rs1048108 | 905 | 1027 | 238 | 854 | 892 | 251 |
| BARD1 | 1140del7 | 2129 | 71 | 1 | 1913 | 59 | 1 |
| BARD1 | rs2229571 | 825 | 1080 | 367 | 817 | 1016 | 345 |
| BARD1 | rs11896262 | 958 | 1020 | 280 | 904 | 964 | 294 |
| BARD1 | rs4673896 | 798 | 1096 | 378 | 770 | 1039 | 367 |
| BAT8 | rs535586 | 2139 | 1908 | 500 | 1930 | 1936 | 507 |
| BAT8 | rs652888 | 2802 | 1527 | 224 | 2611 | 1525 | 225 |
| BRCA1 | rs799917 | 1001 | 1031 | 234 | 939 | 997 | 251 |
| BRCA1 | rs8176199 | 1281 | 857 | 137 | 1207 | 852 | 127 |
| BRCA1 | rs3737559 | 1893 | 362 | 22 | 1833 | 338 | 15 |
| BRCA1 | rs8176166 | 1673 | 553 | 47 | 1580 | 568 | 42 |
| BRCA1 | rs1799950 | 4013 | 511 | 23 | 3879 | 469 | 14 |
| BRCA2 | rs9534174 | 1228 | 2317 | 1006 | 1200 | 2189 | 954 |
| BRCA2 | rs3092989 | 1576 | 628 | 63 | 1504 | 601 | 71 |
| BRCA2 | rs9567552 | 1263 | 875 | 135 | 1187 | 830 | 158 |
| BRCA2 | rs11571686 | 3609 | 893 | 52 | 3432 | 878 | 63 |
| BRCA2 | rs206118 | 3046 | 1328 | 172 | 2943 | 1287 | 132 |
| BRCA2 | rs206119 | 2691 | 1594 | 203 | 2502 | 1486 | 216 |
| BRCA2 | rs4942485 | 1733 | 484 | 34 | 1603 | 414 | 33 |
| BRCA2 | rs206343 | 2791 | 1527 | 225 | 2669 | 1464 | 192 |
| BRCA2 | rs206116 | 1676 | 2145 | 724 | 1621 | 2028 | 705 |
| BRCA2 | rs206115 | 715 | 1083 | 475 | 688 | 1059 | 433 |
| BRCA2 | rs11571742 | 1204 | 907 | 167 | 1205 | 833 | 145 |
| BRCA2 | rs11571789 | 2020 | 235 | 19 | 1909 | 254 | 24 |
| BRCA2 | rs144848 | 2306 | 1892 | 339 | 2183 | 1825 | 331 |
| BRCA2 | rs542551 | 1247 | 829 | 164 | 1187 | 821 | 158 |
| BRCA2 | rs2126042 | 1487 | 669 | 70 | 1453 | 620 | 74 |
| BRCA2 | rs1799955 | 2812 | 1548 | 194 | 2670 | 1492 | 202 |
| BRIP1 | rs11871785 | 958 | 1062 | 247 | 926 | 991 | 264 |
| BRIP1 | rs1557720 | 803 | 1066 | 378 | 792 | 1018 | 346 |
| BRIP1 | rs11652980 | 2004 | 265 | 6 | 1931 | 247 | 4 |
| BRIP1 | rs2191248 | 980 | 995 | 289 | 912 | 976 | 274 |
| BRIP1 | rs2191249 | 1239 | 882 | 156 | 1194 | 826 | 169 |
| BRIP1 | rs2048718 | 655 | 1151 | 458 | 655 | 1063 | 452 |
| BRIP1 | rs16945628 | 1012 | 1008 | 251 | 975 | 938 | 263 |
| BRIP1 | rs4988344 | 1609 | 616 | 53 | 1552 | 585 | 52 |
| BRIP1 | rs4968451 | 1602 | 621 | 53 | 1550 | 586 | 51 |
| BRIP1 | rs2378908 | 1735 | 497 | 45 | 1662 | 496 | 32 |
| BRIP1 | rs9908659 | 892 | 1047 | 327 | 858 | 979 | 327 |
| BRIP1 | rs6504074 | 1229 | 831 | 179 | 1174 | 777 | 182 |
| BRIP1 | rs16945643 | 1958 | 292 | 15 | 1872 | 284 | 15 |
| C17orf37 | rs4252665 | 2108 | 150 | 1 | 1992 | 172 | 0 |
| PERLD1 | rs907089 | 974 | 1025 | 270 | 965 | 969 | 256 |
| PERLD1 | rs2952151 | 1064 | 969 | 243 | 1047 | 922 | 220 |
| PERLD1 | rs1565920 | 1024 | 1014 | 236 | 1000 | 952 | 232 |
| CASP8 | rs1045485 | 3330 | 949 | 81 | 3117 | 828 | 66 |
| CASP8 | rs3769818 | 1171 | 850 | 164 | 1042 | 797 | 162 |
| CASP8 | rs3769824 | 1982 | 193 | 10 | 1839 | 166 | 9 |
| CAT | rs769218 | 1374 | 733 | 124 | 1228 | 717 | 101 |
| CAT | rs7104301 | 1199 | 860 | 150 | 1132 | 775 | 143 |
| CAT | rs1001179 | 1362 | 787 | 113 | 1350 | 710 | 116 |
| CAT | rs1049982 | 2026 | 1963 | 462 | 1904 | 1894 | 416 |
| CAT | rs511895 | 1507 | 2211 | 746 | 1449 | 1997 | 777 |
| CCND1 | rs3862792 | 2052 | 122 | 1 | 1918 | 122 | 1 |
| CCND1 | rs678653 | 1887 | 2036 | 548 | 1699 | 1949 | 559 |
| CCND1 | rs603965 | 635 | 1081 | 478 | 627 | 983 | 428 |
| CCND1 | rs3212891 | 734 | 1123 | 418 | 692 | 1067 | 431 |
| CCND1 | rs602652 | 613 | 1136 | 519 | 612 | 1057 | 502 |
| CCND1 | rs7178 | 3851 | 637 | 26 | 3587 | 613 | 20 |
| CCND1 | rs3212879 | 665 | 1127 | 479 | 614 | 1076 | 483 |
| CCND2 | rs3217926 | 1678 | 2155 | 721 | 1586 | 2121 | 659 |
| CCND2 | rs3217869 | 899 | 1030 | 338 | 808 | 1050 | 325 |
| CCND2 | rs3217936 | 1074 | 961 | 239 | 1004 | 936 | 247 |
| CCND2 | rs3217901 | 764 | 1089 | 423 | 745 | 1074 | 371 |
| CCND2 | rs3217852 | 1304 | 827 | 143 | 1283 | 778 | 128 |
| CCND2 | rs3217862 | 1588 | 626 | 59 | 1511 | 605 | 71 |
| CCND2 | rs3217906 | 1244 | 858 | 172 | 1214 | 814 | 157 |
| CCND2 | rs3217820 | 883 | 1052 | 328 | 842 | 1056 | 283 |
| CCND2 | rs3217933 | 1265 | 852 | 155 | 1226 | 826 | 136 |
| CCND2 | rs3217925 | 1283 | 837 | 141 | 1213 | 825 | 133 |
| CCND2 | rs3217795 | 1828 | 423 | 18 | 1756 | 409 | 21 |
| CCND2 | rs3217916 | 1198 | 904 | 169 | 1144 | 879 | 165 |
| CCND2 | rs3217863 | 1947 | 318 | 8 | 1874 | 310 | 9 |
| CCND2 | rs3217805 | 784 | 1123 | 342 | 812 | 1021 | 330 |
| CCND3 | Rs1410492 | 1216 | 887 | 169 | 1191 | 847 | 146 |
| CCND3 | rs3218110 | 1302 | 818 | 154 | 1216 | 825 | 142 |
| CCND3 | rs9529 | 1164 | 939 | 165 | 1173 | 849 | 161 |
| CCND3 | rs3218114 | 1576 | 635 | 64 | 1490 | 641 | 53 |
| CCND3 | rs3218092 | 1561 | 647 | 68 | 1498 | 636 | 57 |
| CCND3 | Rs2479717 | 1195 | 918 | 154 | 1203 | 835 | 148 |
| CCND3 | Rs1051130 | 706 | 1073 | 493 | 715 | 1015 | 458 |
| CCNE1 | Rs997669 | 1690 | 2146 | 710 | 1492 | 2136 | 739 |
| CCNE1 | rs3218036 | 1077 | 964 | 238 | 987 | 956 | 243 |
| CCNE1 | rs3218076 | 1232 | 869 | 176 | 1170 | 880 | 137 |
| CCNE1 | rs3218038 | 2103 | 169 | 0 | 2027 | 157 | 1 |
| CDK2 | Rs1045435 | 1910 | 342 | 16 | 1838 | 326 | 20 |
| CDK2 | Rs2069408 | 1035 | 960 | 266 | 971 | 964 | 239 |
| CDK4 | rs2270777 | 738 | 1056 | 412 | 630 | 1029 | 383 |
| CDK6 | rs3757823 | 3722 | 788 | 30 | 3510 | 812 | 35 |
| CDK6 | rs2237570 | 3623 | 866 | 64 | 3518 | 794 | 57 |
| CDK6 | rs3731348 | 2026 | 230 | 4 | 1914 | 252 | 3 |
| CDK6 | rs445 | 1823 | 416 | 27 | 1735 | 412 | 24 |
| CDK6 | rs8179 | 1426 | 692 | 109 | 1325 | 724 | 96 |
| CDK6 | rs42046 | 1234 | 875 | 159 | 1141 | 874 | 152 |
| CDK6 | rs3731343 | 617 | 1151 | 493 | 619 | 1083 | 466 |
| CDK6 | rs2079147 | 654 | 1121 | 493 | 623 | 1065 | 493 |
| CDK6 | rs992519 | 1658 | 577 | 37 | 1614 | 512 | 47 |
| CDK6 | rs2285332 | 1261 | 871 | 141 | 1245 | 783 | 151 |
| CDK6 | rs4729049 | 1868 | 379 | 20 | 1803 | 364 | 14 |
| CDK6 | rs8 | 1382 | 783 | 75 | 1349 | 700 | 88 |
| CDK6 | rs2282991 | 3713 | 777 | 43 | 3625 | 693 | 38 |
| CDKN1A | rs3176331 | 1723 | 518 | 34 | 1662 | 486 | 37 |
| CDKN1A | rs3176326 | 1407 | 776 | 85 | 1348 | 730 | 92 |
| CDKN1A | rs2395655 | 846 | 1077 | 353 | 804 | 1013 | 371 |
| CDKN1A | rs6457937 | 1785 | 371 | 21 | 1636 | 364 | 30 |
| CDKN1A | rs762624 | 2429 | 1816 | 307 | 2275 | 1730 | 352 |
| CDKN1A | rs1801270 | 3929 | 519 | 18 | 3674 | 522 | 35 |
| CDKN1A | rs3176336 | 1693 | 2199 | 650 | 1557 | 2047 | 750 |
| CDKN1A | rs3176343 | 2060 | 211 | 5 | 1960 | 219 | 10 |
| CDKN1A | rs3176352 | 2433 | 1707 | 306 | 2236 | 1630 | 333 |
| CDKN1B | rs34329 | 1064 | 983 | 216 | 1061 | 873 | 242 |
| CDKN1B | rs2066827 | 1267 | 796 | 116 | 1190 | 732 | 102 |
| CDKN1B | rs1420023 | 1804 | 417 | 31 | 1750 | 388 | 28 |
| CDKN1B | rs3759216 | 638 | 1154 | 448 | 665 | 1036 | 446 |
| CDKN1B | rs3759217 | 1731 | 514 | 30 | 1696 | 457 | 30 |
| CDKN1B | rs7330 | 1589 | 2178 | 706 | 1451 | 2064 | 707 |
| CDKN1B | rs34330 | 2577 | 1635 | 235 | 2352 | 1616 | 262 |
| CDKN1B | rs3093736 | 2126 | 136 | 3 | 2043 | 123 | 6 |
| CDKN2A | rs3731249 | 2140 | 132 | 2 | 2052 | 122 | 2 |
| CDKN2A | Rs3731222 | 1510 | 538 | 32 | 1463 | 489 | 47 |
| CDKN2A | Rs3217992 | 919 | 1014 | 314 | 869 | 994 | 298 |
| CDKN2A | rs4074785 | 1863 | 390 | 23 | 1817 | 357 | 16 |
| CDKN2A | rs3731197 | 849 | 1074 | 331 | 816 | 1001 | 343 |
| CDKN2A | rs3731257 | 1297 | 822 | 158 | 1208 | 841 | 139 |
| CDKN2A | rs3088440 | 1872 | 365 | 22 | 1833 | 335 | 15 |
| CDKN2A | Rs3731211 | 2298 | 1916 | 322 | 2161 | 1804 | 379 |
| CDKN2A | Rs2811712 | 3664 | 848 | 42 | 3463 | 848 | 65 |
| CDKN2A | Rs3218005 | 1880 | 373 | 20 | 1760 | 393 | 31 |
| CDKN2A | rs3731239 | 1770 | 2174 | 596 | 1845 | 1952 | 561 |
| CDKN2A | Rs3218020 | 1005 | 974 | 266 | 966 | 916 | 266 |
| CDKN2A | rs11515 | 1688 | 553 | 38 | 1598 | 540 | 52 |
| CDKN2B | rs1063192 | 693 | 1134 | 446 | 700 | 1057 | 424 |
| CDKN2B | rs3218012 | 703 | 1117 | 440 | 710 | 1039 | 427 |
| CDKN2B | rs3218009 | 1735 | 512 | 27 | 1660 | 484 | 32 |
| CDKN2C | Rs12855 | 1833 | 418 | 18 | 1798 | 350 | 21 |
| CDKN2C | rs3176459 | 1012 | 1014 | 251 | 946 | 966 | 255 |
| CDKN2D | rs1465701 | 2047 | 219 | 5 | 1958 | 206 | 7 |
| CDKN2D | rs3218222 | 1351 | 783 | 134 | 1254 | 803 | 125 |
| CHEK1 | rs515255 | 1227 | 2300 | 1019 | 1243 | 2158 | 960 |
| CHEK1 | rs565416 | 1112 | 954 | 191 | 1118 | 873 | 184 |
| CHEK1 | rs510443 | 910 | 978 | 295 | 930 | 917 | 280 |
| CHEK1 | rs3731466 | 2148 | 73 | 3 | 2094 | 60 | 2 |
| CHEK1 | rs537046 | 1664 | 564 | 45 | 1620 | 509 | 52 |
| CHEK1 | rs3731438 | 1713 | 504 | 43 | 1640 | 489 | 37 |
| CHEK2 | rs17508019 | 1992 | 270 | 5 | 1886 | 286 | 7 |
| CHEK2 | rs1076807 | 3866 | 633 | 28 | 3770 | 563 | 23 |
| CHEK2 | rs2236141 | 3439 | 1021 | 86 | 3200 | 1086 | 69 |
| CHEK2 | rs2073327 | 1296 | 813 | 152 | 1242 | 790 | 133 |
| CHEK2 | rs9608698 | 1410 | 2188 | 940 | 1249 | 2123 | 956 |
| CHEK2 | rs2347443 | 1554 | 633 | 66 | 1548 | 558 | 62 |
| CHEK2 | rs738722 | 1150 | 938 | 182 | 1129 | 869 | 175 |
| CHEK2 | rs5762760 | 914 | 1045 | 315 | 856 | 1020 | 306 |
| CHEK2 | rs4035540 | 1235 | 872 | 144 | 1198 | 834 | 127 |
| CHEK2 | rs1807609 | 1150 | 928 | 184 | 1130 | 851 | 178 |
| COMT | rs6269 | 773 | 1045 | 368 | 687 | 969 | 372 |
| COMT | rs4680 | 599 | 1052 | 525 | 526 | 982 | 504 |
| CREBBP | rs130021 | 1806 | 2102 | 642 | 1809 | 1999 | 567 |
| CREBBP | rs886528 | 709 | 1158 | 412 | 696 | 1052 | 442 |
| CREBBP | rs9392 | 1341 | 832 | 94 | 1316 | 760 | 106 |
| CREBBP | rs11644593 | 1824 | 426 | 28 | 1743 | 433 | 17 |
| CREBBP | rs8046065 | 1735 | 509 | 33 | 1678 | 475 | 38 |
| CREBBP | rs11076785 | 1006 | 1023 | 249 | 974 | 974 | 241 |
| CRKS | rs4404103 | 1859 | 394 | 22 | 1791 | 379 | 17 |
| CRKS | rs2303315 | 1685 | 551 | 37 | 1656 | 496 | 29 |
| CSTF1 | rs16979884 | 1790 | 373 | 21 | 1645 | 367 | 30 |
| CSTF1 | rs6064389 | 601 | 1110 | 479 | 580 | 980 | 467 |
| CYP19A1 | rs10046 | 620 | 1084 | 466 | 584 | 983 | 471 |
| CYP19A1 | rs11575899 | 923 | 952 | 286 | 843 | 930 | 244 |
| DNMT1 | rs2290684 | 551 | 1117 | 536 | 506 | 1015 | 457 |
| DNMT1 | rs2241531 | 1866 | 264 | 7 | 1779 | 240 | 10 |
| DNMT3A | rs7587636 | 642 | 1132 | 502 | 623 | 1097 | 468 |
| DNMT3A | rs6749992 | 634 | 1111 | 529 | 592 | 1083 | 511 |
| DNMT3A | rs749131 | 699 | 1100 | 431 | 675 | 1061 | 402 |
| DNMT3A | rs7560488 | 794 | 1091 | 389 | 763 | 1053 | 372 |
| DNMT3A | rs752208 | 1408 | 767 | 101 | 1365 | 704 | 121 |
| DNMT3A | rs734693 | 2432 | 1718 | 312 | 2326 | 1587 | 295 |
| DNMT3A | rs2276598 | 1579 | 569 | 54 | 1451 | 537 | 54 |
| DNMT3A | rs2276599 | 2458 | 1703 | 318 | 2351 | 1583 | 289 |
| DNMT3A | rs2289195 | 739 | 1126 | 407 | 745 | 1060 | 380 |
| DNMT3A | rs1465764 | 1894 | 291 | 10 | 1790 | 239 | 7 |
| DNMT3A | rs11677670 | 1476 | 726 | 73 | 1470 | 657 | 62 |
| DNMT3A | rs7581217 | 1958 | 2015 | 578 | 1752 | 2060 | 548 |
| DNMT3A | rs2304429 | 775 | 1106 | 396 | 744 | 1046 | 401 |
| DNMT3A | rs6722613 | 754 | 1107 | 417 | 719 | 1100 | 369 |
| DNMT3B | rs6119954 | 1564 | 641 | 74 | 1472 | 654 | 64 |
| DNMT3B | rs406193 | 3378 | 1089 | 81 | 3367 | 921 | 72 |
| DNMT3B | rs6058897 | 572 | 1125 | 571 | 577 | 1089 | 517 |
| DNMT3B | rs2424932 | 811 | 1092 | 366 | 806 | 1031 | 344 |
| DNMT3B | rs2424928 | 703 | 1111 | 462 | 663 | 1072 | 455 |
| DNMT3B | rs437302 | 1845 | 413 | 21 | 1782 | 381 | 23 |
| DNMT3B | rs992472 | 824 | 1036 | 343 | 767 | 962 | 314 |
| DNMT3B | rs2424913 | 662 | 1059 | 431 | 622 | 1000 | 403 |
| DNMT3B | rs1333469 | 1541 | 2141 | 768 | 1508 | 1964 | 723 |
| E2F5 | rs4150880 | 1317 | 827 | 117 | 1315 | 741 | 107 |
| EGF | rs2237054 | 2008 | 259 | 6 | 1922 | 249 | 10 |
| EHMT1 | rs4526432 | 703 | 1121 | 454 | 683 | 1067 | 440 |
| EHMT1 | rs4634736 | 3575 | 932 | 50 | 3529 | 810 | 44 |
| EHMT1 | rs3125795 | 4032 | 509 | 13 | 3832 | 515 | 26 |
| EHMT1 | rs6559218 | 925 | 1085 | 264 | 901 | 994 | 297 |
| EHMT1 | rs3812497 | 1211 | 885 | 182 | 1182 | 830 | 176 |
| EHMT1 | rs7852475 | 1792 | 2133 | 631 | 1746 | 2018 | 596 |
| C11orf30 | rs2155220 | 691 | 1073 | 440 | 641 | 995 | 400 |
| C11orf30 | rs2513511 | 1690 | 526 | 42 | 1636 | 500 | 36 |
| C11orf30 | rs4245443 | 803 | 1025 | 364 | 730 | 989 | 318 |
| C11orf30 | rs11600501 | 1956 | 187 | 3 | 1822 | 177 | 3 |
| C11orf30 | rs2508755 | 675 | 1114 | 479 | 650 | 1033 | 437 |
| C11orf30 | rs1939469 | 1577 | 558 | 61 | 1436 | 543 | 53 |
| C11orf30 | rs228611 | 2118 | 1953 | 481 | 2003 | 1882 | 470 |
| C11orf30 | rs2508740 | 847 | 1009 | 343 | 757 | 991 | 297 |
| ERBB2 | rs1801201 | 4438 | 108 | 1 | 4294 | 73 | 0 |
| ERBB2 | rs1058808 | 960 | 982 | 238 | 914 | 875 | 234 |
| ERBB2 | rs1810132 | 1022 | 956 | 230 | 967 | 862 | 220 |
| ERBB2 | rs4252596 | 1645 | 511 | 33 | 1550 | 429 | 42 |
| ERBB2 | rs2952155 | 1219 | 839 | 147 | 1159 | 739 | 140 |
| ERBB2 | rs1801200 | 1229 | 791 | 134 | 1131 | 752 | 113 |
| ERBB2 | rs1476278 | 919 | 971 | 267 | 899 | 890 | 247 |
| ESR1 | rs1884051 | 1072 | 998 | 208 | 960 | 1004 | 223 |
| ESR1 | rs3020314 | 2132 | 1970 | 449 | 1870 | 1987 | 500 |
| ESR1 | rs3020317 | 2983 | 1420 | 151 | 2746 | 1427 | 188 |
| ESR1 | rs3020394 | 1083 | 968 | 211 | 941 | 1002 | 229 |
| ESR1 | rs3020396 | 1108 | 956 | 201 | 970 | 987 | 221 |
| ESR1 | rs3020401 | 1110 | 964 | 200 | 974 | 994 | 221 |
| ESR1 | rs3020377 | 1078 | 982 | 213 | 945 | 1001 | 237 |
| ESR1 | rs3020390 | 1083 | 988 | 207 | 951 | 1005 | 231 |
| ESR1 | rs3020416 | 1510 | 649 | 98 | 1400 | 693 | 76 |
| ESR1 | rs2813549 | 1311 | 834 | 127 | 1247 | 796 | 137 |
| ESR1 | rs2234693 | 1318 | 2296 | 934 | 1261 | 2164 | 937 |
| ESR1 | rs4365941 | 2069 | 198 | 7 | 2001 | 182 | 4 |
| ESR1 | rs3020404 | 1143 | 921 | 211 | 1109 | 896 | 181 |
| ESR1 | rs3020405 | 1105 | 971 | 183 | 981 | 975 | 209 |
| ESR1 | rs1801132.2 | 1367 | 805 | 102 | 1302 | 775 | 115 |
| ESR1 | rs3020400 | 1101 | 958 | 196 | 966 | 981 | 217 |
| ESR1 | rs6557178 | 1515 | 687 | 68 | 1418 | 692 | 69 |
| ESR1 | rs9341066 | 1980 | 289 | 6 | 1925 | 253 | 8 |
| ESR1 | rs2347923 | 937 | 1048 | 270 | 893 | 1000 | 279 |
| ESR1 | rs9340835 | 1018 | 1002 | 252 | 951 | 987 | 253 |
| ESR1 | rs3020376 | 1365 | 646 | 230 | 1271 | 660 | 222 |
| ESR1 | rs2982896 | 1404 | 746 | 109 | 1303 | 754 | 102 |
| ESR1 | rs988328 | 1662 | 565 | 52 | 1559 | 576 | 49 |
| ESR1 | rs7766762 | 1721 | 513 | 30 | 1673 | 447 | 34 |
| ESR1 | rs6916218 | 1301 | 817 | 140 | 1208 | 825 | 134 |
| ESR1 | rs746432 | 1847 | 396 | 17 | 1807 | 342 | 22 |
| ESR1 | rs1033181 | 1981 | 284 | 12 | 1910 | 258 | 6 |
| ESR1 | rs3020372 | 1631 | 569 | 71 | 1521 | 608 | 52 |
| ESR1 | rs4583998 | 1040 | 1026 | 207 | 1006 | 955 | 224 |
| ESR1 | rs1543404 | 667 | 1111 | 497 | 640 | 1064 | 477 |
| ESR1 | rs6557168 | 898 | 1037 | 315 | 849 | 1011 | 291 |
| ESR1 | rs9340851 | 2012 | 261 | 6 | 1938 | 236 | 11 |
| ESR1 | rs2747647 | 1816 | 433 | 24 | 1752 | 415 | 21 |
| ESR1 | rs9322340 | 1308 | 848 | 119 | 1269 | 779 | 138 |
| ESR1 | rs9322337 | 2121 | 152 | 4 | 2032 | 155 | 2 |
| ESR1 | rs3798577 | 638 | 1155 | 478 | 616 | 1088 | 477 |
| ESR1 | rs3778610 | 2217 | 61 | 0 | 2134 | 53 | 1 |
| ESR1 | rs2431260 | 1187 | 863 | 175 | 1109 | 821 | 141 |
| ESR1 | rs2747652 | 616 | 1100 | 531 | 592 | 1066 | 492 |
| ESR1 | rs2077647 | 612 | 1177 | 484 | 603 | 1069 | 510 |
| ESR1 | rs1606678 | 1878 | 376 | 16 | 1821 | 337 | 21 |
| ESR1 | rs1062577 | 1970 | 284 | 13 | 1877 | 294 | 8 |
| ESR1 | rs3003925 | 1421 | 779 | 79 | 1370 | 717 | 101 |
| ESR1 | rs9322335 | 1301 | 826 | 152 | 1204 | 859 | 124 |
| ESR1 | ESR1-46 | 1490 | 695 | 91 | 1420 | 671 | 97 |
| ESR1 | rs3020384 | 1268 | 834 | 176 | 1162 | 891 | 137 |
| ESR1 | rs7754762 | 1809 | 447 | 20 | 1727 | 436 | 25 |
| ESR1 | rs9341052 | 1969 | 293 | 15 | 1868 | 309 | 11 |
| ESR1 | rs2175898 | 616 | 1119 | 542 | 591 | 1099 | 501 |
| ESR1 | rs3778099 | 1854 | 405 | 18 | 1738 | 424 | 22 |
| ESR1 | rs9340799 | 1873 | 2048 | 526 | 1681 | 1970 | 521 |
| ESR1 | rs9340821 | 1794 | 454 | 25 | 1739 | 393 | 26 |
| ESR1 | rs9371559 | 1868 | 370 | 23 | 1803 | 354 | 12 |
| ESR1 | rs1709182 | 1796 | 2117 | 607 | 1671 | 2014 | 643 |
| ESR1 | rs9478249 | 806 | 1106 | 363 | 816 | 1029 | 315 |
| ESR1 | rs1514348 | 830 | 1096 | 343 | 748 | 1064 | 372 |
| ESR1 | rs1884054 | 943 | 1058 | 270 | 842 | 1056 | 288 |
| ESR1 | rs3853251 | 1020 | 988 | 258 | 921 | 993 | 263 |
| ESR1 | rs3020407 | 1112 | 962 | 199 | 979 | 987 | 218 |
| ESR1 | rs2144025 | 1698 | 542 | 36 | 1588 | 548 | 46 |
| ESR1 | rs926779 | 1224 | 879 | 173 | 1106 | 919 | 160 |
| ESR1 | rs985191 | 1749 | 509 | 19 | 1640 | 529 | 21 |
| ESR1 | rs2273207 | 1915 | 342 | 9 | 1810 | 353 | 15 |
| ESR1 | rs926777 | 2566 | 1722 | 266 | 2401 | 1703 | 271 |
| ESR1 | rs2813544 | 1370 | 801 | 100 | 1371 | 711 | 101 |
| ESR1 | rs532010 | 850 | 1089 | 309 | 798 | 1027 | 335 |
| ESR1 | rs7382115 | 1587 | 611 | 71 | 1484 | 619 | 73 |
| ESR1 | rs2459107 | 1053 | 981 | 243 | 967 | 982 | 240 |
| ESR1 | rs726282 | 1878 | 385 | 15 | 1748 | 423 | 21 |
| ESR2 | rs1256049 | 1963 | 131 | 0 | 1896 | 128 | 2 |
| ESR2 | rs4986938 | 834 | 1038 | 309 | 771 | 981 | 283 |
| TNFSF6 | rs29087 | 668 | 1073 | 441 | 635 | 977 | 407 |
| TNFSF6 | 76474a>t | 1590 | 545 | 50 | 1461 | 526 | 45 |
| TNFSF6 | rs10458360 | 683 | 1044 | 460 | 635 | 994 | 413 |
| TNFSF6 | rs763110 | 900 | 992 | 316 | 815 | 930 | 282 |
| FBXW7 | -69a>t | 1057 | 920 | 196 | 981 | 850 | 165 |
| GPX1 | rs3448 | 2511 | 1738 | 308 | 2380 | 1665 | 324 |
| GPX4 | rs757229 | 1280 | 2205 | 1020 | 1163 | 2185 | 949 |
| GPX4 | rs4807543 | 2042 | 228 | 3 | 1970 | 209 | 9 |
| GPX4 | rs2075710 | 1379 | 765 | 127 | 1271 | 791 | 115 |
| GPX4 | rs713041 | 1411 | 2193 | 938 | 1327 | 2189 | 846 |
| GPX4 | rs4807542 | 1585 | 604 | 70 | 1469 | 635 | 64 |
| GRB7 | rs11078921 | 983 | 1032 | 254 | 954 | 974 | 250 |
| GRB7 | rs8192704 | 1703 | 533 | 37 | 1679 | 476 | 38 |
| GSR | rs4628224 | 1504 | 662 | 110 | 1440 | 649 | 98 |
| GSR | rs8191009 | 1458 | 723 | 87 | 1352 | 741 | 75 |
| GSR | rs8190973 | 2054 | 220 | 3 | 1972 | 212 | 5 |
| GSR | rs8190969 | 2061 | 211 | 4 | 1973 | 208 | 5 |
| GSR | rs1002149 | 1619 | 594 | 60 | 1530 | 609 | 49 |
| GSR | rs8190924 | 2036 | 219 | 4 | 1963 | 223 | 7 |
| GSR | rs2911678 | 1395 | 767 | 107 | 1306 | 783 | 97 |
| GSR | rs8190951 | 2118 | 153 | 3 | 2013 | 159 | 6 |
| GSR | rs3926402 | 1722 | 2085 | 719 | 1661 | 2040 | 645 |
| GSR | rs2978663 | 1763 | 2081 | 619 | 1696 | 2071 | 598 |
| HDAC2 | rs11391 | 1674 | 470 | 38 | 1557 | 444 | 30 |
| HDAC2 | rs10499079 | 3948 | 569 | 24 | 3801 | 549 | 16 |
| HDAC2 | rs352068 | 1546 | 671 | 57 | 1512 | 613 | 62 |
| HDAC2 | rs352063 | 902 | 1009 | 289 | 841 | 946 | 248 |
| HDAC2 | rs6908846 | 1942 | 314 | 13 | 1886 | 292 | 9 |
| HDAC2 | rs3778216 | 2448 | 1791 | 304 | 2323 | 1722 | 316 |
| HDAC5 | rs228769 | 1420 | 749 | 109 | 1387 | 707 | 95 |
| HDAC5 | rs228757 | 1241 | 886 | 151 | 1224 | 832 | 136 |
| HDAC5 | rs3815076 | 1993 | 280 | 7 | 1915 | 272 | 7 |
| ICAM5 | rs1056538 | 1744 | 2124 | 673 | 1689 | 2037 | 622 |
| ITGB1BP1 | rs7588065 | 762 | 1072 | 335 | 761 | 941 | 344 |
| IGF1 | rs9742718 | 1821 | 356 | 14 | 1697 | 323 | 25 |
| IGF1 | rs6219 | 3706 | 730 | 32 | 3505 | 678 | 46 |
| IGF1 | rs5742694 | 3213 | 1165 | 99 | 3099 | 1045 | 72 |
| IGF1 | rs5742678 | 2605 | 1671 | 271 | 2441 | 1610 | 314 |
| IGF1 | rs6220 | 2406 | 1741 | 314 | 2223 | 1627 | 357 |
| IGF1 | rs1520220 | 3099 | 1251 | 122 | 2878 | 1185 | 155 |
| IGF1 | rs1549543 | 2660 | 1588 | 233 | 2471 | 1482 | 273 |
| IGF1 | rs5742615 | 2215 | 57 | 4 | 2117 | 65 | 5 |
| IGF1 | rs2946834 | 2050 | 1889 | 452 | 1926 | 1824 | 449 |
| IGF1 | rs6214 | 705 | 1130 | 356 | 706 | 985 | 347 |
| IGF1R | rs1863276 | 1829 | 431 | 18 | 1715 | 453 | 17 |
| IGF1R | rs1567811 | 968 | 949 | 229 | 923 | 873 | 220 |
| IGF1R | rs4966036 | 1459 | 722 | 96 | 1370 | 719 | 95 |
| IGF1R | rs1879612 | 898 | 1067 | 306 | 878 | 985 | 315 |
| IGF1R | rs4966021 | 929 | 1031 | 301 | 860 | 1003 | 280 |
| IGF1R | rs8027767 | 790 | 1088 | 398 | 771 | 1036 | 378 |
| IGF1R | rs1568502 | 1262 | 772 | 102 | 1144 | 723 | 97 |
| IGF1R | rs7165875 | 1361 | 789 | 116 | 1281 | 784 | 112 |
| IGF1R | rs2715442 | 1077 | 975 | 224 | 1027 | 911 | 244 |
| IGF1R | IGF1R-10 | 1399 | 692 | 95 | 1293 | 631 | 117 |
| IGF1R | rs1546713 | 696 | 1061 | 392 | 687 | 991 | 340 |
| IGF1R | rs2229765 | 626 | 1092 | 475 | 617 | 1027 | 383 |
| IGF1R | rs8030950 | 1248 | 872 | 154 | 1195 | 831 | 158 |
| IGF2 | rs680 | 1071 | 954 | 160 | 1032 | 844 | 158 |
| IGF2 | rs3741211 | 1779 | 2049 | 687 | 1677 | 2052 | 605 |
| IGF2 | 6815a>t | 1252 | 794 | 133 | 1141 | 753 | 123 |
| IGF2 | 1252t>c | 438 | 828 | 354 | 434 | 707 | 338 |
| IGF2 | IGF2-05 | 1223 | 887 | 161 | 1154 | 852 | 172 |
| IGF2 | IGF2-06 | 974 | 1030 | 250 | 938 | 972 | 262 |
| IGFBP3 | rs2132571 | 1065 | 955 | 193 | 983 | 881 | 172 |
| IGFBP3 | rs2471551 | 1436 | 663 | 88 | 1308 | 646 | 75 |
| IGFBP3 | rs2854744 | 1306 | 2203 | 940 | 1293 | 2092 | 820 |
| IGFBP3 | rs2132572 | 2709 | 1548 | 220 | 2446 | 1519 | 242 |
| IL10 | rs3024496 | 538 | 1111 | 530 | 496 | 1032 | 509 |
| IL10 | rs1800872 | 1338 | 764 | 116 | 1251 | 679 | 115 |
| IL10 | rs1800890 | 743 | 1096 | 346 | 695 | 1003 | 344 |
| IL10 | rs1554286 | 1499 | 614 | 63 | 1385 | 583 | 69 |
| LIG4 | rs3093739 | 1665 | 470 | 40 | 1544 | 468 | 32 |
| LIG4 | rs1805386 | 3086 | 1252 | 127 | 2944 | 1137 | 112 |
| LIG4 | rs3093738 | 1908 | 281 | 7 | 1772 | 240 | 12 |
| LIG4 | rs3093737 | 1754 | 342 | 18 | 1640 | 320 | 12 |
| LIG4 | rs3093772 | 1749 | 470 | 39 | 1676 | 451 | 40 |
| LIG4 | rs3093740 | 2170 | 21 | 2 | 2009 | 24 | 0 |
| LIG4 | rs10131 | 1813 | 366 | 24 | 1672 | 350 | 17 |
| MBD2 | rs1259936 | 977 | 1020 | 280 | 938 | 992 | 257 |
| MBD2 | rs584330 | 1387 | 767 | 102 | 1351 | 721 | 96 |
| MBD2 | rs140691 | 1056 | 995 | 227 | 1022 | 965 | 200 |
| MBD2 | rs10502986 | 1190 | 926 | 159 | 1140 | 867 | 177 |
| MCS5A6 | rs10758440 | 2706 | 1601 | 240 | 2497 | 1589 | 254 |
| MCS5A6 | rs999988 | 2955 | 1396 | 168 | 2810 | 1353 | 181 |
| MCS5A6 | rs2182317 | 3430 | 1045 | 72 | 3425 | 877 | 62 |
| MCS5A6 | rs2381718 | 1163 | 2257 | 1122 | 1103 | 2171 | 1055 |
| MCS5A6 | rs6476643 | 2517 | 1704 | 290 | 2372 | 1657 | 317 |
| MDM2 | rs2279744 | 932 | 959 | 320 | 841 | 1005 | 264 |
| MDM2 | rs1201644 | 620 | 1091 | 542 | 549 | 1101 | 499 |
| MDM2 | rs3730635 | 2008 | 250 | 13 | 1939 | 240 | 8 |
| MDM2 | rs3730581 | 988 | 989 | 301 | 905 | 1031 | 254 |
| MDM2 | rs2291857 | 891 | 1034 | 339 | 829 | 1039 | 302 |
| MDM2 | rs1206251 | 760 | 1099 | 413 | 711 | 1086 | 384 |
| MMP13 | 77a>g | 1057 | 951 | 212 | 978 | 847 | 210 |
| MMP2 | -1306c>t | 2519 | 1658 | 290 | 2368 | 1606 | 247 |
| MMP8 | rs2508383 | 1436 | 729 | 89 | 1433 | 655 | 85 |
| MMP8 | rs11225395 | 735 | 1096 | 437 | 628 | 1105 | 439 |
| MMP8 | rs1276284 | 1991 | 2014 | 533 | 1821 | 2002 | 527 |
| MMP8 | rs7943404 | 763 | 1092 | 420 | 734 | 1070 | 371 |
| MMP8 | rs10895353 | 1894 | 354 | 22 | 1864 | 303 | 23 |
| MMP8 | rs17099436 | 2017 | 249 | 9 | 1963 | 218 | 5 |
| MMP8 | rs1320632 | 1894 | 352 | 15 | 1815 | 335 | 22 |
| MMP8 | rs1940475 | 620 | 1082 | 554 | 528 | 1103 | 534 |
| MMP8 | rs1892886 | 1274 | 847 | 139 | 1140 | 856 | 161 |
| MRE11A | rs13447720 | 1307 | 822 | 140 | 1295 | 771 | 111 |
| MRE11A | rs641936 | 1027 | 1004 | 241 | 946 | 988 | 247 |
| MRE11A | rs11020789 | 1841 | 413 | 25 | 1802 | 369 | 23 |
| MRE11A | rs592943 | 993 | 1037 | 247 | 959 | 957 | 275 |
| MRE11A | rs2508678 | 937 | 1053 | 287 | 890 | 1021 | 280 |
| MRE11A | rs640627 | 1190 | 915 | 170 | 1100 | 913 | 180 |
| MRE11A | rs493982 | 767 | 1099 | 407 | 714 | 1065 | 403 |
| MTHFR | rs1801133 | 1002 | 994 | 245 | 929 | 856 | 248 |
| MUS81 | rs34891773 | 2118 | 158 | 2 | 2056 | 127 | 3 |
| NBS1 | rs14448 | 2031 | 239 | 6 | 1955 | 217 | 11 |
| NBS1 | rs1805839 | 2155 | 114 | 2 | 2066 | 110 | 1 |
| NBS1 | rs6470522 | 1579 | 638 | 58 | 1509 | 614 | 58 |
| NBS1 | rs6998169 | 1852 | 403 | 18 | 1771 | 388 | 21 |
| NBS1 | rs9995 | 2036 | 1993 | 509 | 1946 | 1899 | 513 |
| NBS1 | rs1805787 | 1081 | 954 | 208 | 961 | 860 | 207 |
| NBS1 | rs6470524 | 1058 | 974 | 238 | 987 | 953 | 240 |
| NBS1 | rs13312840 | 1848 | 406 | 17 | 1778 | 377 | 22 |
| NEUROD2 | rs12453682 | 1090 | 937 | 244 | 1021 | 935 | 219 |
| NR1I2 | rs2472671 | 1499 | 657 | 83 | 1397 | 681 | 78 |
| NR1I2 | rs3732357 | 1143 | 905 | 178 | 1064 | 892 | 173 |
| NR1I2 | rs3814055 | 895 | 1047 | 316 | 829 | 1047 | 302 |
| NR1I2 | rs1403526 | 1054 | 998 | 223 | 1037 | 923 | 229 |
| NR1I2 | rs1054190 | 1742 | 494 | 32 | 1633 | 516 | 32 |
| NR1I2 | rs7643645 | 873 | 1064 | 321 | 895 | 964 | 303 |
| NR1I2 | rs3732359 | 1378 | 773 | 104 | 1312 | 751 | 94 |
| NR1I2 | rs13059232 | 1739 | 2163 | 640 | 1725 | 1949 | 676 |
| NUMA1 | rs3750913 | 4327 | 216 | 2 | 4137 | 231 | 3 |
| EP300 | rs5758222 | 959 | 1021 | 291 | 923 | 976 | 285 |
| EP300 | rs1046088 | 2128 | 147 | 4 | 2058 | 129 | 3 |
| EP300 | rs20551 | 1171 | 935 | 169 | 1156 | 878 | 153 |
| EP300 | rs229496 | 1920 | 334 | 21 | 1814 | 360 | 16 |
| EXOSC5 | rs4807 | 1171 | 864 | 150 | 1122 | 763 | 135 |
| PCNXL3 | rs1205259 | 903 | 1010 | 267 | 873 | 891 | 274 |
| PGDS | rs10516950 | 709 | 1136 | 433 | 673 | 1082 | 432 |
| PGR | rs506487 | 922 | 1010 | 262 | 873 | 902 | 258 |
| PGR | rs578938 | 660 | 603 | 152 | 652 | 585 | 125 |
| PGR | rs566351 | 1779 | 2056 | 584 | 1710 | 1892 | 546 |
| PGR | rs1042838 | 3070 | 1119 | 88 | 2803 | 1125 | 101 |
| PGR | rs7116336 | 3759 | 735 | 52 | 3546 | 751 | 51 |
| PGR | rs10895068 | 4005 | 529 | 14 | 3857 | 493 | 12 |
| PGR | rs660149 | 2353 | 1855 | 334 | 2356 | 1677 | 318 |
| PGR | rs11571171 | 2119 | 1962 | 453 | 2094 | 1804 | 433 |
| PGR | rs492457 | 1181 | 851 | 155 | 1151 | 754 | 132 |
| PGR | rs500760 | 2662 | 1572 | 213 | 2438 | 1576 | 208 |
| PPARBP | rs11655550 | 1516 | 665 | 92 | 1419 | 673 | 84 |
| PPARBP | rs9904919 | 1260 | 837 | 177 | 1187 | 825 | 167 |
| PPARBP | rs6503513 | 1481 | 694 | 99 | 1420 | 662 | 103 |
| PPP1CB | 459a>g | 750 | 1055 | 345 | 740 | 947 | 303 |
| PPP1R1B | rs879606 | 1520 | 607 | 71 | 1443 | 582 | 64 |
| PPP1R1B | rs1874228 | 1210 | 860 | 175 | 1159 | 831 | 165 |
| PPP1R2P1 | PPP1R2P1-01 | 937 | 1026 | 289 | 885 | 993 | 285 |
| PTGDS | rs908839 | 634 | 1135 | 493 | 594 | 1080 | 499 |
| PTGDS | rs11787588 | 1213 | 885 | 166 | 1211 | 820 | 144 |
| PTGDS | rs10781530 | 1104 | 941 | 229 | 1019 | 953 | 212 |
| PTGES | rs2302821 | 1902 | 353 | 12 | 1872 | 298 | 14 |
| PTGES | rs10739757 | 1808 | 436 | 17 | 1710 | 435 | 26 |
| PTGES | rs7872802 | 1752 | 495 | 27 | 1667 | 489 | 36 |
| PTGES | rs10988484 | 3190 | 1251 | 104 | 3085 | 1136 | 108 |
| PTGES | rs3844048 | 1071 | 990 | 213 | 1041 | 928 | 205 |
| PTGES | rs4636306 | 1531 | 684 | 56 | 1480 | 630 | 73 |
| PTGES | rs4837404 | 957 | 1029 | 277 | 929 | 974 | 275 |
| PTGES | rs7855291 | 1247 | 878 | 137 | 1229 | 819 | 120 |
| PTGES | rs10448290 | 3794 | 724 | 26 | 3605 | 698 | 44 |
| PTGIS | rs6095541 | 1561 | 644 | 65 | 1461 | 643 | 73 |
| PTGIS | rs477627 | 3248 | 1161 | 137 | 3119 | 1154 | 87 |
| PTGIS | rs6090996 | 2856 | 1502 | 184 | 2824 | 1343 | 190 |
| PTGIS | rs6095543 | 1590 | 619 | 55 | 1501 | 611 | 71 |
| PTGIS | rs556731 | 4123 | 408 | 11 | 3978 | 361 | 13 |
| PTGIS | rs1393343 | 1373 | 799 | 103 | 1365 | 705 | 117 |
| PTGIS | rs501908 | 1831 | 417 | 29 | 1736 | 425 | 27 |
| PTGIS | rs693649 | 1440 | 741 | 91 | 1429 | 674 | 79 |
| PTGIS | rs707528 | 572 | 1159 | 537 | 562 | 1055 | 558 |
| PTGIS | rs6090990 | 1261 | 841 | 139 | 1205 | 818 | 141 |
| PTGIS | rs508757 | 1467 | 711 | 89 | 1417 | 659 | 95 |
| PTGIS | rs6012696 | 1969 | 299 | 10 | 1876 | 305 | 11 |
| PTGIS | rs8183919 | 2272 | 1908 | 373 | 2125 | 1803 | 429 |
| PTGIS | rs544187 | 1882 | 370 | 14 | 1846 | 328 | 9 |
| PTGIS | rs5602 | 1168 | 2264 | 1040 | 1094 | 2076 | 1106 |
| PTGS1 | rs1330344 | 1413 | 748 | 112 | 1395 | 683 | 112 |
| PTGS1 | rs4836887 | 1702 | 543 | 33 | 1665 | 492 | 31 |
| PTGS1 | rs10306108 | 1952 | 306 | 13 | 1867 | 302 | 12 |
| PTGS1 | rs10306122 | 1969 | 285 | 10 | 1902 | 271 | 9 |
| PTGS1 | rs10306194 | 1594 | 610 | 53 | 1578 | 543 | 49 |
| PTGS2 | rs689467 | 1978 | 293 | 9 | 1881 | 303 | 11 |
| PTGS2 | rs20424 | 2183 | 91 | 0 | 2118 | 74 | 0 |
| PTGS2 | rs5277 | 1562 | 639 | 56 | 1573 | 550 | 51 |
| PTGS2 | rs4648310 | 2119 | 153 | 4 | 2062 | 119 | 4 |
| PTPRJ | rs1503185 | 1541 | 589 | 66 | 1426 | 563 | 56 |
| PTPRJ | rs4752894 | 786 | 1073 | 413 | 737 | 1046 | 402 |
| PTPRJ | rs2904315 | 1020 | 961 | 210 | 937 | 888 | 209 |
| PTPRJ | rs1566729 | 3050 | 1307 | 148 | 2972 | 1226 | 132 |
| PTPRJ | rs7122335 | 2879 | 1407 | 193 | 2669 | 1420 | 201 |
| RAD50 | rs2706338 | 1451 | 716 | 80 | 1401 | 673 | 99 |
| RAD50 | rs17772583 | 1282 | 857 | 132 | 1200 | 828 | 139 |
| RAD50 | rs6884762 | 2136 | 123 | 2 | 2042 | 128 | 2 |
| RAD50 | rs2237060 | 1465 | 2288 | 785 | 1448 | 2110 | 768 |
| RAD50 | rs17772565 | 2079 | 185 | 4 | 1984 | 192 | 4 |
| RAD51 | RAD51-08 | 2005 | 261 | 10 | 1939 | 242 | 11 |
| RAD51 | rs2619681 | 1551 | 653 | 57 | 1515 | 611 | 58 |
| RAD51 | rs1801320 | 1995 | 257 | 14 | 1911 | 236 | 13 |
| RAD51 | rs7402012 | 1555 | 662 | 57 | 1526 | 608 | 54 |
| RAD51 | rs11852786 | 607 | 1119 | 551 | 555 | 1067 | 563 |
| RAD51 | rs12442560 | 1467 | 2192 | 835 | 1402 | 2055 | 854 |
| RAD51C | rs16943716 | 2814 | 1535 | 202 | 2718 | 1439 | 211 |
| RAD51C | rs9916423 | 1997 | 274 | 3 | 1916 | 258 | 10 |
| RAD51C | rs304267 | 973 | 1027 | 261 | 971 | 942 | 261 |
| RAD51C | rs304270 | 857 | 1080 | 336 | 858 | 1010 | 321 |
| RAD51C | rs17822908 | 1417 | 746 | 92 | 1336 | 703 | 116 |
| RAD52 | rs3748521 | 571 | 1154 | 455 | 623 | 1059 | 447 |
| RAD52 | rs6413436 | 809 | 1123 | 316 | 822 | 1013 | 328 |
| RAD52 | rs4987208 | 2200 | 75 | 0 | 2111 | 76 | 1 |
| RAD52 | rs4987207 | 2226 | 51 | 0 | 2138 | 54 | 0 |
| RAD52 | rs6489766 | 944 | 1014 | 317 | 917 | 975 | 291 |
| RAD52 | rs11226 | 615 | 1123 | 441 | 645 | 1058 | 448 |
| RAD52 | rs11571497 | 2230 | 40 | 0 | 2142 | 46 | 0 |
| RAD52 | rs11571452 | 1621 | 591 | 52 | 1599 | 525 | 55 |
| RAD52 | ra1051669 | 1287 | 830 | 149 | 1200 | 832 | 157 |
| RAD52 | rs10774474 | 1483 | 676 | 91 | 1441 | 660 | 86 |
| RAD52 | rs11571379 | 611 | 1103 | 526 | 606 | 1049 | 504 |
| RAD52 | rs11571409 | 610 | 1113 | 464 | 639 | 1010 | 447 |
| RAD54L | rs10789488 | 1202 | 892 | 173 | 1156 | 880 | 145 |
| RAD54L | rs9793263 | 766 | 1099 | 383 | 725 | 1062 | 370 |
| RAD54L | rs12410307 | 1862 | 391 | 17 | 1789 | 365 | 24 |
| RAD54L | rs2295464 | 3474 | 1010 | 67 | 3352 | 938 | 81 |
| RAD54L | rs12146051 | 1085 | 961 | 226 | 1034 | 946 | 205 |
| RAD54L | rs28363196 | 1770 | 453 | 38 | 1671 | 460 | 34 |
| RAD54L | rs17102087 | 1802 | 453 | 21 | 1738 | 427 | 23 |
| RB1 | rs9535032 | 1134 | 884 | 189 | 1021 | 832 | 180 |
| RB1 | rs1981434 | 1171 | 905 | 188 | 1115 | 865 | 191 |
| RB1 | rs2854344 | 3738 | 659 | 29 | 3635 | 543 | 23 |
| RB1 | rs520342 | 2511 | 1647 | 292 | 2316 | 1608 | 256 |
| RB1 | rs4151540 | 1220 | 874 | 179 | 1146 | 863 | 168 |
| RB1 | rs4151551 | 1779 | 395 | 23 | 1659 | 360 | 17 |
| RB1 | rs198610 | 2081 | 129 | 3 | 1895 | 131 | 3 |
| RB1 | rs4151636 | 2092 | 181 | 4 | 1997 | 186 | 5 |
| RB1 | rs4151611 | 4160 | 387 | 7 | 3955 | 390 | 8 |
| RB1 | rs198580 | 4187 | 251 | 3 | 4003 | 186 | 4 |
| RB1 | rs2854345 | 1489 | 625 | 77 | 1311 | 632 | 66 |
| RB1 | rs399413 | 1157 | 855 | 164 | 1033 | 821 | 164 |
| RB1 | rs2227311 | 1645 | 521 | 49 | 1552 | 451 | 41 |
| RELA | rs1049728 | 1997 | 266 | 11 | 1903 | 277 | 5 |
| RELA | rs10896027 | 925 | 1062 | 286 | 907 | 975 | 302 |
| RELA | rs11568300 | 929 | 1042 | 284 | 883 | 1025 | 258 |
| RELA | rs732072 | 1969 | 293 | 9 | 1894 | 280 | 9 |
| RELA | rs2306365 | 1688 | 536 | 27 | 1624 | 489 | 42 |
| PRDM2 | rs2495061 | 1273 | 804 | 134 | 1158 | 731 | 120 |
| PRDM2 | rs2235515 | 2724 | 1606 | 227 | 2532 | 1575 | 265 |
| PRDM2 | rs1203638 | 1359 | 787 | 129 | 1281 | 801 | 109 |
| PRDM2 | rs2495090 | 2769 | 1591 | 189 | 2658 | 1501 | 204 |
| PRDM2 | rs2495051 | 1269 | 838 | 167 | 1213 | 833 | 134 |
| PRDM2 | rs1203651 | 889 | 1035 | 291 | 806 | 950 | 291 |
| PRDM2 | rs2235512 | 2651 | 1590 | 226 | 2433 | 1516 | 253 |
| PRDM2 | rs1046331 | 1621 | 534 | 44 | 1473 | 526 | 41 |
| SETDB1 | rs4970986 | 958 | 1051 | 262 | 945 | 984 | 257 |
| SETDB1 | rs11204744 | 1240 | 885 | 153 | 1207 | 831 | 146 |
| SHBG | rs858518 | 1450 | 2262 | 830 | 1473 | 2100 | 780 |
| SHBG | rs6259 | 1725 | 526 | 28 | 1708 | 447 | 33 |
| SHBG | rs858521 | 872 | 1064 | 323 | 869 | 1008 | 297 |
| SHBG | rs272428 | 692 | 1111 | 471 | 703 | 1066 | 417 |
| SHBG | rs1799941 | 1246 | 873 | 158 | 1184 | 840 | 166 |
| SHBG | rs13894 | 1941 | 309 | 12 | 1874 | 291 | 9 |
| SHBG | rs858524 | 1568 | 2177 | 803 | 1407 | 2148 | 811 |
| SIPA1 | rs3741378 | 1640 | 524 | 30 | 1526 | 472 | 40 |
| SIPA1 | rs746429 | 917 | 992 | 261 | 816 | 950 | 232 |
| SIPA1 | rs931127 | 623 | 1099 | 470 | 623 | 970 | 412 |
| SOD1 | rs2070424 | 3869 | 576 | 20 | 3663 | 536 | 16 |
| SOD1 | rs202445 | 1485 | 662 | 58 | 1380 | 591 | 70 |
| SOD1 | rs4998557 | 3540 | 901 | 59 | 3314 | 879 | 43 |
| SOD2 | rs2842958 | 1402 | 691 | 119 | 1297 | 636 | 108 |
| SOD2 | rs1799725 | 1140 | 2135 | 1138 | 1057 | 2105 | 1025 |
| SOD2 | rs2758339 | 575 | 1085 | 546 | 514 | 1040 | 488 |
| STARD3 | rs3817160 | 1377 | 767 | 122 | 1304 | 730 | 119 |
| SULT1E1 | rs1534672 | 511 | 783 | 347 | 515 | 758 | 312 |
| SULT1E1 | rs3775775 | 3672 | 747 | 43 | 3361 | 807 | 43 |
| SULT1E1 | rs1881668 | 2398 | 1757 | 318 | 2167 | 1720 | 310 |
| SULT1E1 | rs1238574 | 2060 | 205 | 5 | 1939 | 219 | 7 |
| SULT1E1 | rs4149533 | 1021 | 909 | 224 | 952 | 838 | 188 |
| SULT1E1 | rs3775770 | 1089 | 884 | 200 | 1019 | 827 | 180 |
| SULT1E1 | rs4149529 | 2146 | 1943 | 418 | 2066 | 1826 | 405 |
| SULT1E1 | rs3736599 | 3649 | 781 | 37 | 3357 | 817 | 40 |
| SULT1E1 | rs4149525 | 1673 | 544 | 45 | 1619 | 519 | 32 |
| AURKA | rs2273535 | 2720 | 1515 | 211 | 2519 | 1445 | 219 |
| AURKA | rs1047972 | 1518 | 599 | 62 | 1409 | 582 | 44 |
| AURKA | rs732417 | 3890 | 619 | 26 | 3808 | 512 | 26 |
| AURKA | rs8173 | 1216 | 870 | 159 | 1177 | 834 | 158 |
| STS | rs2017591 | 790 | 1095 | 361 | 781 | 968 | 394 |
| STS | rs1000900 | 1175 | 852 | 159 | 1113 | 755 | 140 |
| STS | rs2024159 | 1141 | 933 | 199 | 1132 | 874 | 181 |
| TBXAS1 | rs3779134 | 1771 | 467 | 37 | 1681 | 465 | 37 |
| TBXAS1 | rs41728 | 1749 | 493 | 26 | 1675 | 459 | 44 |
| TBXAS1 | rs2267701 | 1669 | 548 | 31 | 1602 | 520 | 47 |
| TBXAS1 | rs2267681 | 771 | 1084 | 404 | 764 | 1044 | 367 |
| TBXAS1 | rs740150 | 1507 | 674 | 93 | 1411 | 697 | 77 |
| TBXAS1 | rs2107901 | 1578 | 622 | 64 | 1528 | 566 | 67 |
| TBXAS1 | rs1557967 | 1455 | 733 | 85 | 1397 | 698 | 92 |
| TBXAS1 | rs10487665 | 1878 | 381 | 15 | 1821 | 355 | 16 |
| TBXAS1 | rs2286196 | 1474 | 704 | 89 | 1397 | 708 | 72 |
| TBXAS1 | rs194149 | 1187 | 884 | 194 | 1131 | 872 | 172 |
| TBXAS1 | rs41723 | 1579 | 639 | 55 | 1524 | 610 | 54 |
| TBXAS1 | rs2267692 | 964 | 1011 | 292 | 906 | 968 | 295 |
| TBXAS1 | rs41727 | 3727 | 780 | 38 | 3532 | 748 | 66 |
| TBXAS1 | rs3823715 | 1048 | 971 | 254 | 956 | 973 | 256 |
| TBXAS1 | rs6971207 | 2228 | 50 | 0 | 2127 | 59 | 2 |
| TBXAS1 | rs11973494 | 1385 | 765 | 120 | 1383 | 688 | 112 |
| TBXAS1 | rs2284205 | 1615 | 595 | 64 | 1545 | 587 | 53 |
| TBXAS1 | rs10487667 | 1280 | 832 | 160 | 1170 | 852 | 154 |
| TBXAS1 | rs7810415 | 573 | 1172 | 503 | 558 | 1074 | 534 |
| TBXAS1 | rs1003816 | 1827 | 415 | 20 | 1741 | 400 | 36 |
| TBXAS1 | rs193948 | 760 | 1098 | 409 | 699 | 1055 | 415 |
| TBXAS1 | rs2267706 | 1358 | 782 | 118 | 1269 | 781 | 118 |
| TBXAS1 | rs3779130 | 956 | 1026 | 290 | 885 | 1005 | 289 |
| TBXAS1 | rs2286197 | 880 | 1054 | 319 | 785 | 1064 | 317 |
| TBXAS1 | rs2190087 | 1325 | 821 | 117 | 1255 | 806 | 114 |
| TGFB1 | rs1982073 | 1724 | 2163 | 620 | 1602 | 2056 | 675 |
| TIEG2 | -415a>g | 666 | 1057 | 469 | 608 | 1025 | 397 |
| TIEG2 | Q62R | 1731 | 440 | 27 | 1599 | 417 | 27 |
| TP53 | rs17881013 | 1797 | 438 | 39 | 1700 | 452 | 34 |
| TP53 | rs17887200 | 2053 | 217 | 5 | 1972 | 203 | 6 |
| TP53 | rs17884663 | 865 | 1069 | 342 | 833 | 1022 | 336 |
| TP53 | rs17886760 | 2067 | 204 | 5 | 1987 | 197 | 7 |
| TP53 | rs1042522 | 1177 | 854 | 166 | 1108 | 770 | 148 |
| TP53 | rs1625895 | 1622 | 520 | 55 | 1548 | 449 | 48 |
| TXN | rs1410051 | 2500 | 1636 | 295 | 2358 | 1564 | 262 |
| TXN | rs4135208 | 983 | 947 | 240 | 917 | 874 | 217 |
| TXN | rs4135172 | 1909 | 347 | 19 | 1811 | 348 | 16 |
| TXN | rs4135179 | 2768 | 1545 | 233 | 2743 | 1364 | 262 |
| TXN | rs4135215 | 1594 | 575 | 68 | 1556 | 563 | 56 |
| TXN | rs2301241 | 771 | 1097 | 371 | 717 | 1069 | 359 |
| TXN | rs2418076 | 1215 | 831 | 176 | 1069 | 816 | 147 |
| TXN | rs4135211 | 1889 | 302 | 13 | 1716 | 311 | 10 |
| TXN | rs4135192 | 964 | 1022 | 227 | 926 | 878 | 214 |
| TXN | rs2776 | 538 | 1082 | 523 | 538 | 1015 | 477 |
| TXN | rs4135225 | 1904 | 2041 | 474 | 1789 | 1895 | 486 |
| TXN | rs997392 | 2095 | 82 | 2 | 1947 | 80 | 0 |
| TXN | rs4135165 | 3755 | 763 | 34 | 3545 | 781 | 42 |
| TXN | rs4135168 | 1196 | 809 | 167 | 1128 | 747 | 131 |
| TXN | rs1049927 | 1614 | 515 | 47 | 1530 | 472 | 37 |
| TXN2 | rs2281082 | 1498 | 689 | 74 | 1441 | 663 | 76 |
| TXN2 | rs8139906 | 1537 | 678 | 60 | 1469 | 651 | 70 |
| TXN2 | rs8140110 | 1865 | 379 | 13 | 1766 | 374 | 19 |
| TXNRD1 | rs10861201 | 1349 | 796 | 125 | 1293 | 771 | 120 |
| TXNRD1 | rs4964779 | 1780 | 457 | 34 | 1727 | 424 | 35 |
| TXNRD1 | rs4964778 | 3048 | 1342 | 159 | 2940 | 1280 | 143 |
| TXNRD1 | rs4564401 | 1958 | 302 | 15 | 1863 | 314 | 12 |
| TXNRD2 | rs732262 | 3759 | 756 | 39 | 3594 | 742 | 32 |
| TXNRD2 | rs3788314 | 1347 | 2275 | 927 | 1242 | 2163 | 969 |
| TXNRD2 | rs5748469 | 1988 | 2029 | 514 | 1838 | 1979 | 533 |
| TXNRD2 | rs1548357 | 2329 | 1829 | 383 | 2170 | 1795 | 388 |
| TXNRD2 | rs2073752 | 2350 | 1841 | 328 | 2327 | 1681 | 333 |
| TXNRD2 | rs3788306 | 2210 | 1905 | 429 | 2065 | 1875 | 411 |
| TXNRD2 | rs2020917 | 2351 | 1814 | 355 | 2234 | 1720 | 371 |
| TXNRD2 | rs4485648 | 2896 | 1461 | 179 | 2850 | 1323 | 195 |
| TXNRD2 | rs2073750 | 1420 | 744 | 107 | 1339 | 740 | 101 |
| TXNRD2 | rs740603 | 647 | 1123 | 487 | 619 | 1085 | 457 |
| TXNRD2 | rs756661 | 1313 | 2306 | 927 | 1348 | 2170 | 847 |
| VEGF | rs699947 | 544 | 1054 | 541 | 498 | 1012 | 505 |
| VEGF | rs1570360 | 1006 | 917 | 227 | 926 | 901 | 207 |
| VEGF | rs2010963 | 988 | 936 | 245 | 962 | 872 | 210 |
| VEGF | rs3025039 | 1576 | 495 | 36 | 1509 | 468 | 39 |
| XRCC2 | rs3218536 | 3639 | 711 | 34 | 3590 | 610 | 32 |
| XRCC2 | rs3218417 | 1257 | 871 | 136 | 1230 | 801 | 142 |
| XRCC2 | rs3218384* | 1084 | 683 | 104 | 1062 | 650 | 119 |
| XRCC2 | rs3218499 | 1381 | 793 | 100 | 1354 | 714 | 108 |
| XRCC2 | rs3218374 | 628 | 1143 | 505 | 593 | 1092 | 502 |
| XRCC2 | rs3218416 | 2067 | 198 | 4 | 1986 | 190 | 5 |
| XRCC2 | rs3218400 | 1844 | 402 | 25 | 1765 | 391 | 17 |
| XRCC2 | rs2106776 | 1419 | 2145 | 839 | 1367 | 2032 | 810 |
| XRCC2 | rs3218382 | 1894 | 353 | 4 | 1849 | 319 | 1 |
| XRCC3 | rs861530 | 1116 | 927 | 212 | 1055 | 898 | 193 |
| XRCC3 | rs1799796 | 1998 | 1959 | 461 | 1954 | 1857 | 392 |
| XRCC3 | rs861539 | 897 | 1048 | 317 | 845 | 1024 | 310 |
| XRCC3 | rs861544 | 1090 | 950 | 233 | 1033 | 930 | 219 |
| XRCC3 | rs861529 | 2143 | 133 | 2 | 2065 | 121 | 4 |
| XRCC3 | rs1799794 | 1474 | 706 | 83 | 1407 | 695 | 69 |
| XRCC3 | rs861542 | 2167 | 105 | 1 | 2091 | 96 | 3 |
| ZNFN1A3 | rs10445308 | 620 | 1140 | 518 | 593 | 1086 | 498 |
| ZNFN1A3 | rs907091 | 600 | 1055 | 559 | 563 | 1002 | 528 |

* SNPs without dbSNP reference given alternative designstion. Sequence details for these are available from authors on request
